# Prolonged Exposure to Oxaliplatin during HIPEC Improves Effectiveness in a Preclinical Micrometastasis Model

**DOI:** 10.3390/cancers14051158

**Published:** 2022-02-24

**Authors:** Nick Seyfried, Can Yurttas, Markus Burkard, Benedikt Oswald, Alexander Tolios, Franziska Herster, Joseph Kauer, Tarkan Jäger, Ingmar Königsrainer, Karolin Thiel, Markus Quante, Hans-Georg Rammensee, Sascha Venturelli, Matthias Schwab, Alfred Königsrainer, Stefan Beckert, Markus W. Löffler

**Affiliations:** 1 Department of General, Visceral and Transplant Surgery, University Hospital Tübingen, Hoppe-Seyler-Str. 3, 72076 Tübingen, Germany; nick.seyfried@tum.de (N.S.); ingmar.koenigsrainer@lkhf.at (I.K.); karolin.thiel@med.uni-tuebingen.de (K.T.); markus.quante@med.uni-tuebingen.de (M.Q.); alfred.koenigsrainer@med.uni-tuebingen.de (A.K.); stefan.beckert@sbk-vs.de (S.B.); markus.loeffler@uni-tuebingen.de (M.W.L.); 2 Interfaculty Institute for Cell Biology, Department of Immunology, University of Tübingen, Auf der Morgenstelle 15, 72076 Tübingen, Germany; benedikt.ac.oswald@gmail.com (B.O.); franziska.herster@rbk.de (F.H.); joseph.kauer@med.uni-heidelberg.de (J.K.); rammensee@uni-tuebingen.de (H.-G.R.); 3 Department of Surgery, Klinikum Rechts der Isar, Technical University of Munich (TUM), Ismaninger Str. 22, 81675 Munich, Germany; 4 Institute of Nutritional Sciences, Department of Nutritional Biochemistry, University of Hohenheim, Garbenstr. 30, 70599 Stuttgart, Germany; markus.burkard@uni-hohenheim.de (M.B.); sascha.venturelli@uni-hohenheim.de (S.V.); 5 Department of Blood Group Serology and Transfusion Medicine, Medical University of Vienna, Währinger Gürtel 18-20, 1090 Vienna, Austria; alexander.tolios@meduniwien.ac.at; 6 Center for Physiology and Pharmacology, Institute of Vascular Biology and Thrombosis Research, Medical University of Vienna, Schwarzspanierstraße 17A, 1090 Vienna, Austria; 7 Center for Medical Statistics, Informatics and Intelligent Systems, Institute of Artificial Intelligence, Medical University of Vienna, Spitalgasse 23, 1090 Vienna, Austria; 8 Robert Bosch Center for Tumor Diseases (RBCT), Robert Bosch Hospital, Auerbachstr. 110, 70376 Stuttgart, Germany; 9 German Cancer Consortium (DKTK) and German Cancer Research Center (DKFZ) Partner Site Tübingen, 72076 Tübingen, Germany; matthias.schwab@ikp-stuttgart.de; 10 Clinical Collaboration Unit Translational Immunology, German Cancer Consortium (DKTK), Department of Internal Medicine, University Hospital Tübingen, Otfried-Müller-Str. 10, 72076 Tübingen, Germany; 11 Department of Hematology, Oncology, and Rheumatology, University Hospital Heidelberg, Im Neuenheimer Feld 410, 69120 Heidelberg, Germany; 12 Department of Surgery, Paracelsus Medical University, Müllner Hauptstraße 48, 5020 Salzburg, Austria; ta.jaeger@salk.at; 13 Department of General, Visceral and Thoracic Surgery, Landeskrankenhaus Feldkirch, Carinagasse 47, 6800 Feldkirch, Austria; 14 Cluster of Excellence iFIT (EXC2180) ‘Image-Guided and Functionally Instructed Tumor Therapies’, University of Tübingen, 72076 Tübingen, Germany; 15 Department of Vegetative and Clinical Physiology, Institute of Physiology, University of Tübingen, Wilhelmstr. 56, 72074 Tübingen, Germany; 16 Department of Clinical Pharmacology, University Hospital Tübingen, Auf der Morgenstelle 8, 72076 Tübingen, Germany; 17 Dr. Margarete Fischer-Bosch-Institute of Clinical Pharmacology, Auerbachstr. 112, 70376 Stuttgart, Germany; 18 Departments of Pharmacy and Biochemistry, University of Tübingen, Auf der Morgenstelle 15, 72076 Tübingen, Germany; 19 Department of General and Visceral Surgery, Schwarzwald-Baar Hospital, Klinikstr. 11, 78052 Villingen-Schwenningen, Germany

**Keywords:** PRODIGE 7 trial, peritoneal metastasis, peritoneal carcinomatosis, colorectal cancer, micrometastasis model

## Abstract

**Simple Summary:**

Absence of survival benefits when adding hyperthermic intraperitoneal chemotherapy (HIPEC) with oxaliplatin to cytoreductive surgery in peritoneal metastasis from colorectal cancer has recently been shown in the randomized controlled PRODIGE 7 trial. We therefore aimed to investigate the effects of this treatment modality in a preclinical micrometastasis model. Cancer cells were incubated with either patient samples obtained during HIPEC procedures or with defined oxaliplatin-containing solutions prepared according to clinically established HIPEC protocols. Our results demonstrate a limited effectiveness of short-term HIPEC in simulations with oxaliplatin to eliminate micrometastases, although we used platinum-sensitive cell lines for our model. Since these results are in line with findings from current research, our studies might offer further convincing evidence and potential explanations for HIPEC futility observed in clinical application.

**Abstract:**

Cytoreductive surgery combined with hyperthermic intraperitoneal chemotherapy (HIPEC) was considered a promising treatment for patients with peritoneal metastasis from colorectal cancer. However, the recently published randomized controlled PRODIGE 7 trial failed to demonstrate survival benefits through the addition of short-term oxaliplatin-based HIPEC. Constituting a complex multifactorial treatment, we investigated HIPEC in a preclinical model concerning the elimination of minimal tumor residues, thereby aiming to better understand the size of effects and respective clinical trial results. Patient samples of peritoneal perfusates obtained during HIPEC treatments and oxaliplatin-containing solutions at clinically relevant dosages, conforming with established HIPEC protocols, were assessed regarding their ability to eliminate modelled ~100 µm thickness cancer cell layers. Impedance-based real-time cell analysis and classical end-point assays were used. Flow cytometry was employed to determine the effect of different HIPEC drug solvents on tumor cell properties. Effectiveness of peritoneal perfusate patient samples and defined oxaliplatin-containing solutions proved limited but reproducible. HIPEC simulations for 30 min reduced the normalized cell index below 50% with peritoneal perfusates from merely 3 out of 9 patients within 72 h, indicating full-thickness cytotoxic effects. Instead, prolonging HIPEC to 1 h enhanced these effects and comprised 7 patients’ samples, while continuous drug exposure invariably resulted in complete cell death. Further, frequently used drug diluents caused approximately 25% cell size reduction within 30 min. Prolonging oxaliplatin exposure improved effectiveness of HIPEC to eliminate micrometastases in our preclinical model. Accordingly, insufficient penetration depth, short exposure time, and the physicochemical impact of drug solvents may constitute critical factors.

## 1. Introduction

The combined use of cytoreductive surgery (CRS) and hyperthermic intraperitoneal chemotherapy (HIPEC) for colorectal cancer (CRC) peritoneal metastasis has been supported by a leadoff randomized controlled trial (RCT) in 2003 showing prolonged overall survival for this treatment compared to palliative chemotherapy alone [1]. For decades this combined treatment approach has been in use for peritoneal metastases of different cancers [2]. The theoretical rationale of HIPEC is the elimination of residual tumor cells remaining after CRS through the locoregional administration of chemotherapy, which is justified by the assumption that a compartmental effect caused by the so-called peritoneal–plasma barrier prevents the penetration of intravenous drugs into the abdominal space [3]. The most frequent indication for this treatment has been peritoneal metastasis originating from ovarian cancer or CRC as well as pseudomyxoma peritonei. Accordingly, CRS and HIPEC has recently been shown to improve overall survival in a large cohort study in patients with pseudomyxoma peritonei [4] and can be considered the standard of care in this rare malignant syndrome [5]. In spite of long-standing practice, evidence from RCTs was lacking that could confirm survival benefits for adding HIPEC treatment to CRS alone. Only in 2018 an RCT demonstrated an increase in overall survival through adding 90-min cisplatin-based HIPEC to surgery in selected patients suffering from peritoneal metastasis that originated from platinum-sensitive ovarian cancers [6]. Of note, HIPEC treatment remained very heterogeneous over years [2,7]. In this context, the necessity of uniform and comparable treatment protocols has been rarely addressed, although this has been done for HIPEC with oxaliplatin (OX) in the treatment of CRC [8]. In contrast, attempts of standardization are commonplace for the surgical approach of CRS in peritoneal metastasis [9] and a required learning curve for improvement is well-established [10,11]. Amongst the numerous protocols in use, one of the most frequently applied drugs for peritoneal metastases in CRC is OX [7,12]. However, the recently published PRODIGE 7 study failed to show a survival benefit for the addition of 30-min OX-based HIPEC compared to surgery alone [13]. This multicenter RCT had used 460 mg OX (i.e., 230 µg/mL ≙ 579 µM OX) for open HIPEC (coliseum technique) and 360 mg OX (i.e., 180 µg/mL ≙ 453 µM OX) for closed HIPEC, diluted in dextrose solvent with each 2 L of perfusate filled into the abdomen. Since this outcome was unexpected, many discussions ensued as to why the HIPEC procedure performed so surprisingly poor [14,15,16]. Remarkably, basic and/or pharmacological research that may have shed more light in this context was unavailable. It should be acknowledged that CRS and HIPEC constitute a complex compound treatment with many variables, hence the single contribution of each of the components remains poorly dissected [17].

For this reason, we were interested to investigate how different OX-containing solutions would perform under simulated HIPEC conditions in a ~100 µm thick micrometastasis model. Already in previous work we had established an impedance-based real-time cell analysis (RTCA) assay, enabling continuous monitoring of cancer cells. Hereby, a thorough characterization of patient perfusate samples taken during HIPEC procedures was performed [18]. At this time, we had used respective OX-containing samples diluted with 50% serum-containing cell culture medium and left them incubated with OAW42 ovarian cancer cells. This assay unanimously showed an elimination of the exposed cell layer within 72 h. The aim of the present study was now to assess HIPEC treatment in a preclinical model to separately characterize its effects within the multimodal therapy approach. To this end, we incubated a ~100 µm thick layer of platinum-sensitive tumor cells at 42 °C for 30 min or 60 min with well-defined sample materials previously obtained during HIPEC treatment of patients. We also used OX at clinically relevant concentrations diluted in different solvents, conforming with established HIPEC protocols. This also encompassed the exact OX dosages and diluents that have been reported for the PRODIGE 7 trial [13].

## 2. Materials and Methods

### 2.1. Patient Samples

Studies on materials sampled during patient treatment were approved by the institutional review board at Tübingen University (project number: 367/2013BO1). All patients gave their written informed consent before study inclusion. Patient characteristics and treatment details can be assessed elsewhere [18]. Drug solvent circulated through the abdomen of patients was collected prior to addition of OX (0 min) as well as OX-containing samples taken at 5, 10, 15, 20, 25, and 30 min after starting the HIPEC procedure. For patient 8 and 9, samples were only obtained in 10-min intervals. Samples were stored at −80 °C until usage. Cellular debris and other impurities were cleared by centrifugation (5 min at 13,000× *g*) before use.

### 2.2. Impedance-Based Real-Time Cell Analysis (RTCA)

Materials and methods were used as established before [18], with slight modifications. As described previously, platinum-sensitive OAW42 cells (European Collection of Authenticated Cell Cultures, Salisbury, UK) were grown under appropriate conditions, seeded and used as a model system to monitor effects of OX in real-time [18]. In brief, cells were grown in cell culture medium (DMEM; Dulbecco’s Modified Eagle Medium, high glucose; Gibco/Life Technologies, Carlsbad, CA, USA, adding 10% fetal calf serum (FCS); Sigma-Aldrich/Merck Life Science, St. Louis, MO, USA; 100 U/mL penicillin G; PAA, Pasching, Austria; and 100 µg/mL streptomycin; PAA). Cell cultures were periodically tested for mycoplasma using commercially available polymerase chain reaction (PCR) kits (Minerva Biolabs, Berlin, Germany). An RTCA device (xCELLigence SP; Roche, Grenzach-Wyhlen, Germany) was employed as previously described [18]. After a calibration of the device with 100 µL DMEM (blank values), 5 × 10^4^ OAW42 cells/ well were seeded into dedicated 96-well plates (E-plate 96; ACEA, San Diego, CA, USA) and left to adhere for 24 h. Subsequently, fluid was discarded from wells and adherent cells were washed once with warmed Dulbecco’s phosphate-buffered saline (PBS; Gibco Life Technologies, Carlsbad, CA, USA). Then, either 200 µL of peritoneal perfusate obtained during HIPEC from patients or defined concentrations of OX (Oxaliplatin-GRY/ oxaliplatin 5 mg/mL; Teva, Petach Tikwa, Israel/ Fresenius Kabi, Bad Homburg, Germany; dose range: 5.6–230 µg/mL ≙ 14–579 µM OX) diluted in peritoneal dialysis fluid (PDS; Physioneal 40 Glucose 2.27% *m*/*v*; Baxter, Deerfield, IL, USA) or dextrose 5% in water (D5W; Glucosteril 5%; Fresenius Kabi) were added. Likewise, adequate controls were employed, including 1% (*v*/*v*) Triton X-100 (Sigma) as a positive control (lysis control for dead cells) and negative controls with either PDS or DMEM only. HIPEC conditions were subsequently simulated by incubating cells for 30 min or 60 min at 42 °C in an ambient air incubation shaker (Infors, Bottmingen, Switzerland) with slight movement (50 rotations per min; rpm). Afterwards, liquids were discarded by flicking and cells were washed twice in PBS and then cultivated in appropriate medium. All samples were analyzed at least in duplicates and impedance was measured continuously in 15-min intervals. Technical errors and outliers were removed. For inter-experiment comparability, the cell index was set to 1 immediately before simulated HIPEC treatment (normalized cell index; nCI). Results were analyzed using RTCA software (V. 1.2.1). GraphPad prism software (V. 7.01; GraphPad software Inc., La Jolla, CA, USA) was used for presentation of results. In addition, biorender software was used for visualizations (www.biorender.com). Findings of multiple experiments were combined, when adequate.

### 2.3. CellTiter-Blue^®^ (CTB) Cell Viability Assay

To confirm effects detected by RTCA on cell viability, CTB Cell Viability Assay (Promega, Mannheim, Germany) was performed. OAW42 and HT29 cells were seeded in 24-well plates (3.15 × 10^5^ cells) in a volume of 500 µL in triplicates and incubated overnight at 37 °C and 5% CO_2_ in a humidified atmosphere. To mimic different HIPEC treatment conditions, DMEM was discarded, cells were washed with PBS and then incubated for 30 min at 42 °C under ambient air conditions with OX (at concentrations of 45, 90, 180, and 230 µg/mL; ≙ 113, 227, 453, and 579 µM OX) diluted either in D5W, PDS, or DMEM (control). Following treatment, solutions were discarded and replaced by 500 µL of fresh DMEM after washing with PBS and incubated for 72 h (37 °C; 5% CO_2_). In parallel, OX-spiked DMEM remained on the cells to verify OX toxicity on OAW42 and HT29 cells after 72 h continuous exposure (37 °C; 5% CO_2_). As positive controls, cell death was induced by lysing cells with 1% (*v*/*v*) Triton X-100 (Roth, Karlsruhe, Germany) for 10 min immediately prior to CTB staining. To determine cell viability, 100 µL of assay reagent were added and gently mixed. Fluorescence measurement was performed 1 h after incubation (37 °C; 5% CO_2_) for each cell line with the Synergy HT microtiter plate reader (BioTek Instruments Inc., Winooski, VT, USA; record fluorescence; excitation wavelength: 530/25 and emission wavelength: 590/35, adjusted sensitivity: 35). CTB assays were repeated in 3 independent experiments.

### 2.4. Sulforhodamine B (SRB) Cytotoxicity Assay

OAW42 and HT29 cells were seeded in 24-well plates (3.15 × 10^5^ cells/ well) in a volume of 500 µL as triplicates and incubated overnight (37 °C; 5% CO_2_). To mimic HIPEC treatment, medium was discarded, and cells were washed with PBS and then incubated for 30 min at 42 °C under ambient air conditions with OX diluted either in D5W, PDS, or DMEM (see OX concentrations given above). After treatment, solutions were discarded and replaced by 500 µL of fresh DMEM after washing with PBS and incubated for 72 h (37 °C; 5% CO_2_). In a parallel experiment, OX-spiked DMEM remained on the cells to verify OX effects on OAW42 and HT29 cells after 72 h exposure (37 °C; 5% CO_2_). As positive control, cell death was induced by lysing cells with 1% (*v*/*v*) Triton X-100 (Roth, Karlsruhe, Germany) for 10 min immediately prior to SRB staining. Finally, growth inhibition was evaluated by SRB assay. In brief, medium was discarded, and each well was washed once with ice-cold PBS and fixed with 10% trichloroacetic acid (TCA) for 30 min at 4 °C. After washing with tap water, cells were dried at 40 °C overnight. Then proteins were stained for 10 min with SRB reagent (0.4% (*w*/*v*) in 1% (*v*/*v*) acetic acid; CAS 3520-42-1, Sigma-Aldrich) and after removing unbound dye with tap water followed by 1% (*v*/*v*) acetic acid, dried again at 40 °C. Protein-bound dye was resolved with 10 mM Tris base (pH 10.5). After 10 min incubation at room temperature, optical density was measured in triplicates (80 µL volume/ well) in 96-well plates with a Synergy HT microtiter plate reader (BioTek Instruments; measurement wavelength 550 nm, reference wavelength 620 nm). Data represent the mean of optical density values related to DMEM treated control cells. SRB assays were repeated in 3 independent experiments.

### 2.5. Microscopy

In order to obtain a serial dilution, OAW42 cells were seeded at different numbers of 12.5 × 10^3^, 20 × 10^3^, 25 × 10^3^, 35 × 10^3^, and 50 × 10^3^ cells/ well (96-well flat bottom plate) and incubated in 200 µL DMEM. After 24 h of cell culture, cells were carefully washed with PBS and subsequently treated with fixation buffer (BioLegend, San Diego, CA, USA) for 10 min at room temperature, carefully washed again and kept at 4 °C prior to measurements. Experiments were performed twice. The thickness of cell layers was obtained measuring z-stacks on a Nikon Ti Eclipse microscope (by an unbiased observer) using 10× magnification. The analysis was performed with the NIS-Elements (Nikon, Tokyo, Japan) or ImageJ software V. 1.52 h. 

### 2.6. Flow Cytometry

OAW42 cells were seeded in 96-well plates (1 × 10^5^ cells/ well) in either 200 µL of DMEM, PDS, or D5W and kept for 30 min or 60 min at 42 °C. Immediately prior to flow cytometric analysis, 7-amino-actinomycin D viability staining solution (7-AAD; BioLegend) was added to each well at a final concentration of 600 ng/mL. Dead cells (7-AAD-positive) as well as doublets were excluded. Forward scatter area (FSC-A) served as an indicator for cell size. Samples were analyzed using a FACSCanto II (BD Biosciences, Heidelberg, Germany) and data analysis was performed using FlowJo_V9 software (FlowJo LCC, Ashland, OR, USA). Flow cytometric analysis of cell size under HIPEC conditions was repeated in independent experiments.

## 3. Results

### 3.1. Monitoring HIPEC Effects in a Micrometastasis Model in Real-Time by Impedance Assessment

**Figure 1:**
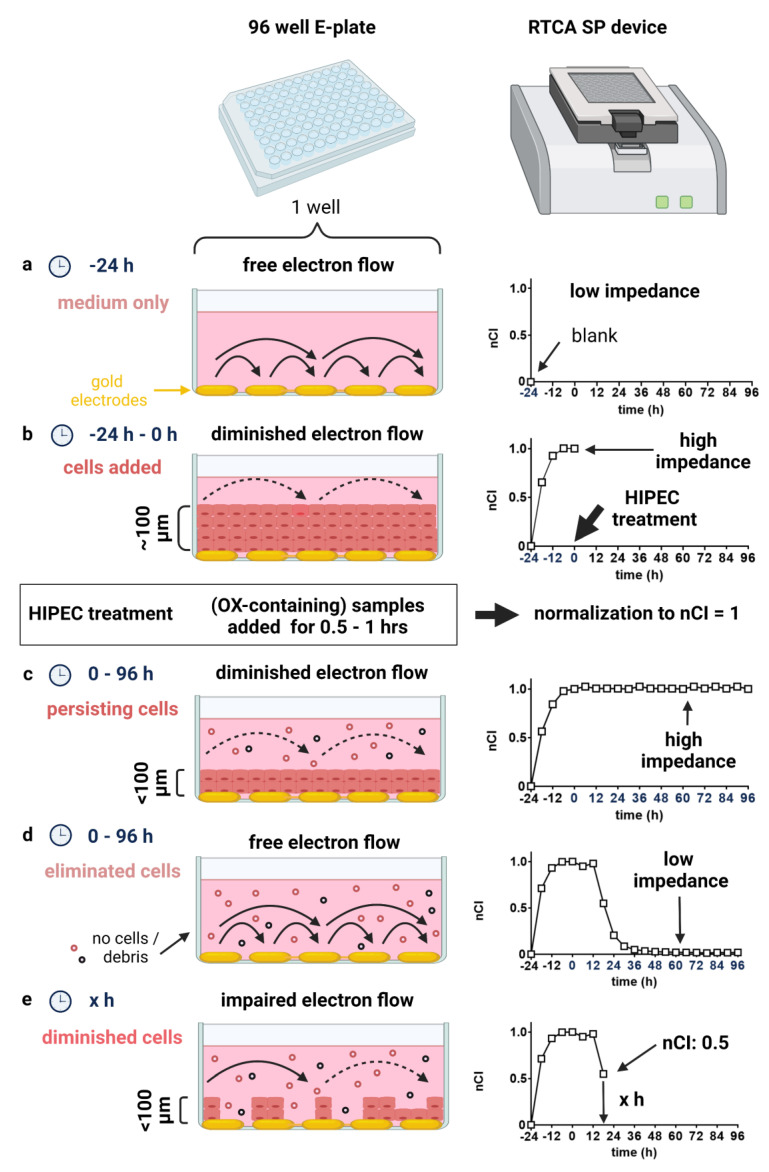
Background on the used micrometastasis model and readout of the used real-time impedance-based cell analysis (RTCA) assay. (**a**) The experiment in a 96-well E-plate is started 24 h before the planned HIPEC treatment. Blank values with only cell culture medium are measured. Under these conditions, electrons can flow freely between the gold electrodes located at the well bottom (baseline; low impedance). (**b**) Subsequently, cells are added to each well and left to attach for 24 h forming a ~100 µm thick cell layer (impedance increases to reach a plateau, since electron flow is heavily impaired through the isolation effects of the added cells). At this point, before HIPEC simulation (**b**), impedance is normalized to 1 (nCI = 1) to allow for comparability between independent experiments. (**c**) HIPEC simulation is performed by incubating cells with OX-containing solutions for 30 min or 60 min at 42 °C with slight movement. Afterwards samples are removed, cells washed and supplemented with fresh medium. Measurement is continued for 4 days. (**d**) If the complete full cell layer is affected, and cells become penetrable in full thickness, while only debris remains, electrons can flow freely, and impedance decreases to baseline values. (**e**) If cells persist and isolation effects impairing free electron flow remain, impedance diminishes depending on intact cells left. An nCI = 0.5 specifies the value when the initial impedance has been bisected and can be determined as a function of time. The thickness of the seeded cells was assessed and the linearity of thickness according to cell numbers seeded per well established (Figure S1). For a relatable scale, the edge length of a grain of salt is about 300 µm.

The 2-dimensional (2D) micrometastasis model as established here aims to recreate the clinical conditions prevailing during HIPEC in a laboratory setting. Not only are cells exposed to hyperthermia and realistic drug dosages but also exposure time and respective solvents can be modelled with either patient samples or OX-containing solutions prepared appropriately in this preclinical model. Of note, the used RTCA system allows continuous monitoring of cell elimination and addresses the clinically relevant question of effectiveness in peritoneal micrometastases after optimal CRS (Figure 1). For this reason, our model system used a cell layer with defined thickness, requiring a penetration depth of about 100 µm for measurable effects (Figure S1).

The RTCA readout is generated by measuring electron flow between an array of electrodes located at the bottom of each well in a specific 96-well E-Plate (Figure 1a). An intact cell layer isolates the electrodes and thereby impairs electron flow. Accordingly, impedance increases, which is indicated by a rising cell index (Figure 1b,c). This effect is proportional to the cell number and morphology of cells covering the electrodes at the well bottom (Figure 1e) [19]. In our model, relevant measurable effects require full thickness defects of the cell layer (decline in cell index Figure 1d). An nCI value of 0.5 specifies that the initially prevailing impedance measured at normalization has been bisected.

### 3.2. Modelling HIPEC with Patient Samples Displays Heterogeneous Effectivity (by RTCA Assay) in a Micrometastasis Model

**Figure 2:**
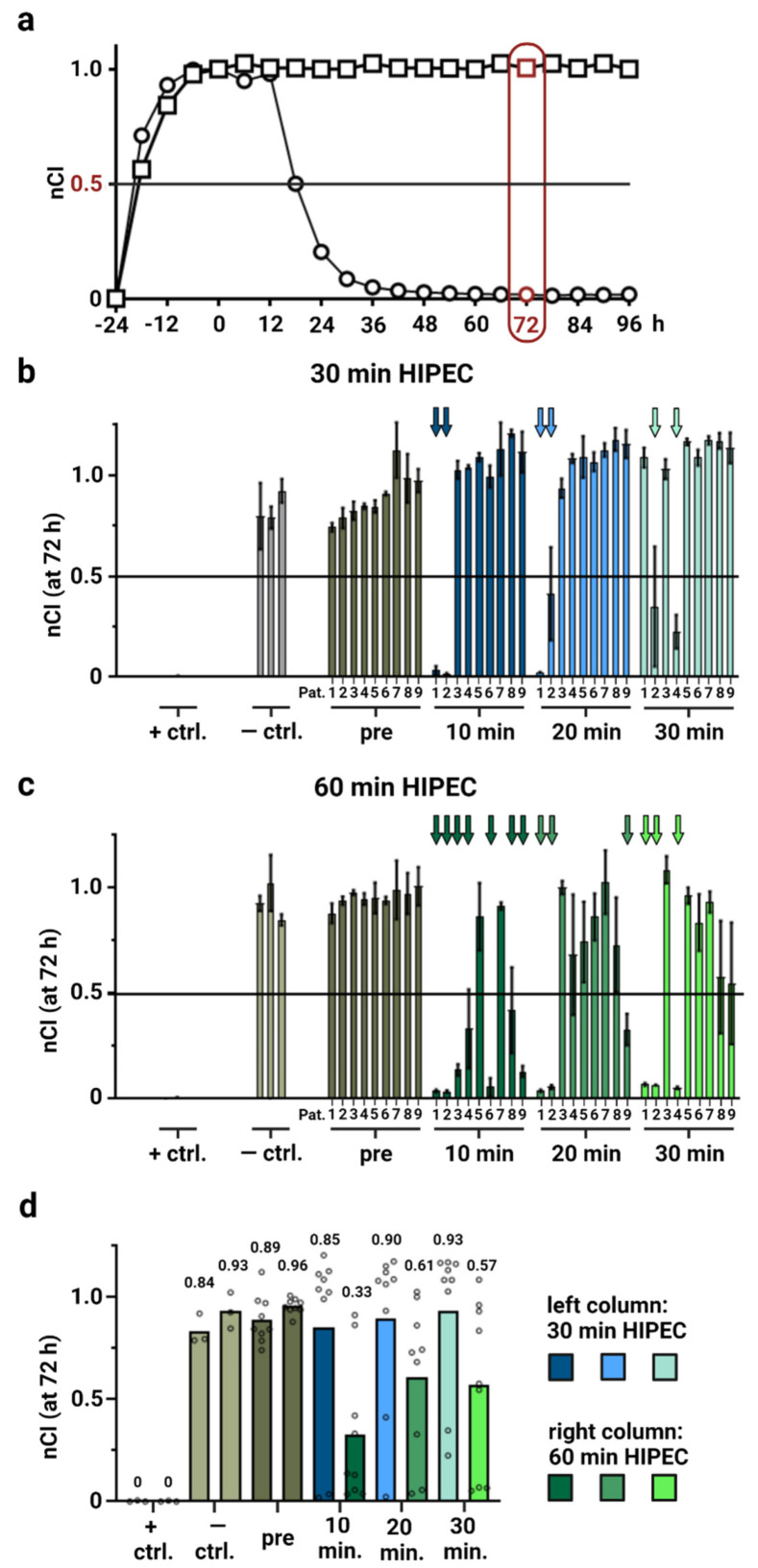
HIPEC simulations using patient samples (RTCA assay). (**a**) Exemplary impedance readings in 12-h intervals. The displayed time point 72 h after HIPEC simulation is marked by a red border. (**b**,**c**) Simulated HIPEC (42 °C at 50 rpm shaking) was performed, either for 30 min (**b**) or for 60 min (**c**) with 5 × 10^4^ OAW42 cells per well. (**d**) Means of replicate values shown in (**b**,**c**) are annotated as dots and respective overall mean values of different patient materials are shown as a bar plot and annotated (left bars/ blue colors: 30 min exposure in HIPEC simulations; right bars/ green colors: 60 min exposure in HIPEC simulations). Due to the large variance and partly bimodal data distribution, any statistical significance testing was omitted and means chosen as a measure of central tendency. X-axis: positive control (+ ctrl.) with Triton X-100, negative control (− ctrl.) (light grey coloration) and HIPEC solutions obtained from patients (Pat.) 1–9 each: samples before/pre (dark grey coloration), 10 min (dark blue/ dark green coloration), 20 min (medium blue/ medium green coloration), and 30 min (light blue/ light green coloration) after adding OX to the HIPEC circuit during patient treatment. Y-axis: nCI determined at 72 h since beginning of measurements after HIPEC treatment. Each colored arrow marks an average decrease in nCI (72 h) below 0.5 (black horizontal line). Depiction of mean values with standard deviation, number of replicates: 2–5.

First, we used sample materials that were obtained during OX-based HIPEC from 9 patients and that have been extensively characterized previously [18]. As shown before, the label-free RTCA assay enables monitoring cells continuously with good temporal resolution. However, now we intended to model HIPEC conditions more precisely and exposed ovarian cancer cells (OAW42) to respective aliquots from HIPEC patient samples for 30 min or 60 min at 42 °C (50 rpm shaking) to simulate respective conditions, subsequently washing cells and extending cell culture with fresh culture medium and regularly assessing nCI values (Figure 2a).

Previously published experiments have shown that these exact OX-containing samples invariably eliminated the seeded cells, when continuously exposing them for 72 h to HIPEC perfusates, even when diluting them through supplementation of 50% medium [18].

In our modified HIPEC model, most aliquots from the previously characterized patient samples [18] proved insufficient to effectively eliminate the cancer cells in our micrometastasis model within 72 h, when HIPEC was simulated for 30 min. Strikingly, in 6 out of 9 investigated patients, none of the obtained OX-containing samples tested was able to reduce impedance below 50% of initial values (nCI < 0.5) within 3 days following HIPEC simulation (Figure 2b). Prolonging exposure time to 1 h considerably improved cytotoxic effects but did not prove generally effective, lowering nCI < 0.5 when using samples from 7 out of 9 patients (Figure 2c). Observed effects were also stronger in those samples that were taken earlier after HIPEC initiation during clinical patient treatment. Further, using the overall means from different patient samples and comparing the different conditions to each other (Figure 2d) revealed clear differences between 30 min and 60 min of OX-based HIPEC simulation. When considering effects after 30 min exposure, both controls and HIPEC perfusates sampled at different time points showed comparable mean nCI readings ranging from 0.84 to 0.96 at 72 h following the treatment. In contrast, prolonging the procedure to 60 min resulted in mean nCI readings of 0.33 with OX-containing HIPEC perfusates sampled in patients after 10 min as well as decreasing mean nCI values to 0.61 (20 min) and 0.57 (30 min) for respective samples obtained later. Although it has to be mentioned that the source data partly show a bimodal distribution as well as a large variance (see also Figure 2c), this evaluation supports improved effects with increased HIPEC duration as well as underscoring patient individual differences.

**Figure 3:**
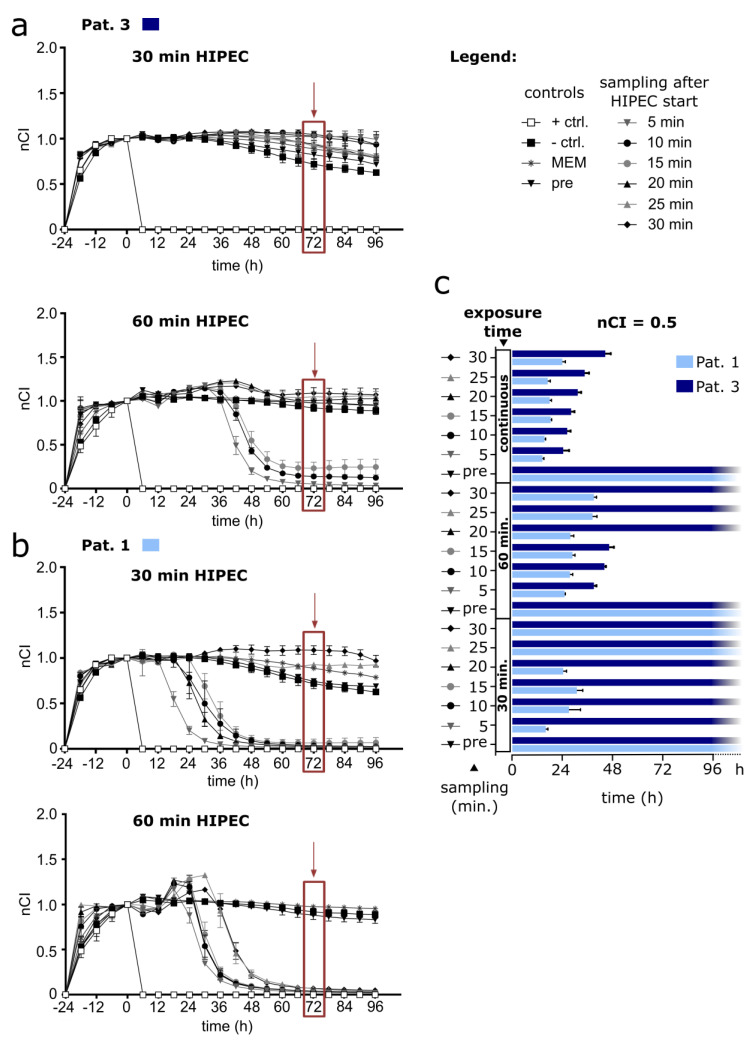
HIPEC simulations for 30 min or 60 min using patient samples (RTCA assay) (**a**,**b**) Simulated HIPEC (42 °C at 50 rpm shaking) was performed for 30 min (upper graph) and 60 min (lower graph) with 5 × 10^4^ OAW42 cells per well. Positive control (+ ctrl.) with Triton X-100, negative control (− ctrl.) with PDS (peritoneal dialysis solution), MEM (cell culture medium), and HIPEC solutions obtained from patient (Pat.) 3 (**a**) and Pat. 1 (**b**). Samples: before (pre), 5, 10, 15, 20, 25, and 30 min after adding OX to the HIPEC circuit during clinical patient treatment. *Y*-axis: nCI determined until 96 h after restarting measurements following HIPEC treatment. (**c**) Duration until nCI = 0.5 was reached after 30 min or 60 min simulated HIPEC or by continuous incubation (nota bene: dilution with 50% MEM) with respective samples. Depiction of mean values with standard deviation, number of replicates: 2–4. Respective RTCA readings with HIPEC sample materials obtained from Pat. 2 and Pat. 4–9 are provided as Supplementary Materials (Figures S2–S15).

The differences in our micrometastasis model for the perfusates sampled in patients at several different time points during HIPEC and used for short-term (30 min) and prolonged (60 min) HIPEC simulations are striking. Looking at the RTCA results from perfusate samples in 2 exemplary patients, we can discern in patient 3 (Figure 3a) that with none of the OX-containing perfusates the exposed cell layer could be effectively eliminated following 30 min of simulated HIPEC, as shown by persistently elevated impedance readings. Prolonging the treatment duration to 1 h could rescue these effects and showed a decrease below nCI = 0.5 for the samples obtained during the first 15 min of clinical HIPEC treatment in this patient, whereas the other samples (20–30 min) proved ineffective. In contrast, patient 1 already showed a respective decrease of nCI to baseline values after 30 min of simulated HIPEC for all samples obtained during the first 20 min of the HIPEC procedure in the clinic (Figure 3b). Here, a prolonged exposure of cells to HIPEC samples over 1 h showed impedance decreasing to baseline values in all OX-containing perfusates tested.

For a comparison of aliquots from the same sample materials of the 2 presented patients, contrasting both 30 min and 60 min of simulated HIPEC to continuous exposure, we annotated the time points when nCI = 0.5 was reached in an overview chart (Figure 3c; patient samples are aliquots from the materials previously characterized and data of continuous incubation were previously published by Löffler et al. [18]).

On a side note, the perfusate used for continuous exposure of cells had to be diluted with medium (50%), since this is inevitably required to sustain cell culture. Respective samples therefore contain only half of the OX concentrations compared to those samples used for 30 min and 60 min of simulated HIPEC (Figure 3c). Still, continuous exposure to OX-containing HIPEC perfusates unanimously led to nCI decreases below 0.5. This was usually reached within 48 h with a slight waning effect over time for perfusates sampled later during HIPEC in patients. A complete dataset with results from the other patients is provided in the Supplementary Materials (Figures S2–S15). Further, it has been established that only 0.05 μM OX is sufficient to kill 50% of directly exposed OAW42 cells (LC_50_) within 72 h [20], whereas usual OX concentrations used during HIPEC are above 90 µM and reach up to 579 µM according to the PRODIGE 7 protocol [13].

### 3.3. Short-Term HIPEC with OX-Containing Solutions Prepared According to Clinically Established Protocols Proves Ineffective in Modelled Micrometastases (RTCA Assay)

Next, we prepared defined OX-containing solutions that comprised accurate amounts of OX in different diluents, aiming to recreate the clinical conditions prevailing during HIPEC as authentically as possible. To this end, we used a concentration range of OX, encompassing the final drug dosages according to most established HIPEC protocols [16], explicitly including those used in the PRODIGE 7 study [13] and conforming with previously published HIPEC models [21].

We assessed the effects of respective OX concentrations diluted either in D5W or PDS in our 100 µm thickness micrometastasis model after simulating the HIPEC procedure for 30 min. Subsequently, drugs were removed, and cells were washed and cultured in medium for the long-term.

**Figure 4:**
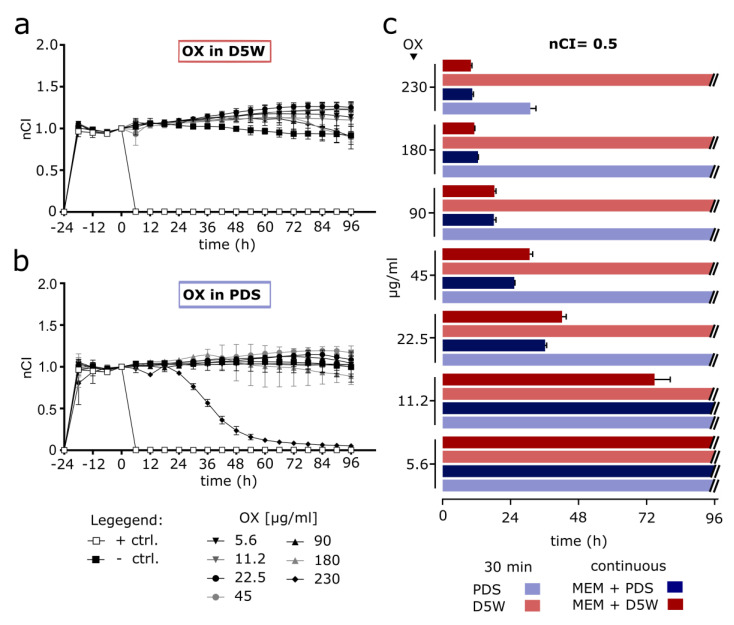
HIPEC simulation for 30 min using prepared OX-containing solutions (RTCA assay) (**a**,**b**) Simulated HIPEC (42 °C and 50 rpm shaking) was performed for 30 min with 5 × 10^4^ OAW42 cells per well. Positive control (+ ctrl.) with Triton X-100, negative control (− ctrl.) and prepared solutions with specified OX concentrations in dextrose 5% (D5W) (**a**) or peritoneal dialysis solutions (PDS) (**b**). Y-axis: nCI determined until 96 h (h) after restarting measurements following HIPEC treatment. (**c**) Duration until nCI = 0.5 was reached after 30 min simulated HIPEC or when continuously incubated (dilution with 50% cell culture medium (MEM)) with respective samples. Depiction of mean values with standard deviation, number of replicates: 2–3. Respective RTCA readings with continuous OX exposure are provided as Supplementary Materials (Figures S17 and S18).

Here, we did not observe any substantial treatment induced decrease in nCI after 30 min of OX-based HIPEC using either D5W (Figure 4a) or PDS (Figure 4b) as a solvent within 96 h following treatment, with the exception of the highest dosage of 230 µg/mL OX diluted in PDS. Prolongation to 60 min simulated HIPEC showed effective for 180 µg/mL and 230 µg/mL OX when diluted with PDS (see Supplementary Materials: Figure S16). However, 60 min HIPEC exposure with OX diluted in D5W proved technically unfeasible due to recurring detachment of the treated cell layer.

Again, when exposing cells continuously to OX at the specified concentrations diluted in cell culture medium (here doubled concentrations were used to account for 50% dilution with medium), this intervention proved effective to reduce nCI below 0.5 for a broad concentration range. For example, concentrations of 11.2–230 µg/mL OX proved effective when diluted in D5W and for 22.5–230 µg/mL in PDS. For the dose range of 22.5–45 µg/mL OX it appears that dilution in PDS reached nCI = 0.5 slightly faster than with D5W. Further higher drug concentrations seemed associated with reaching nCI = 0.5 earlier than when using a lower drug concentration. However, measurable effects still took several hours to appear.

### 3.4. Confirming RTCA Results with Prepared OX-Containing Solutions by Classical End-Points Assays

To confirm the RTCA results previously obtained, we performed 2 different well-established conventional end-point assays. The fluorometric assay CTB is based on the conversion of resazurin to resorufin, occurring only in living cells [22], and a cytotoxicity assay using SRB, which binds stoichiometrically to proteins under mild acidic conditions [23]. Cells were densely seeded to reach comparable conditions as used previously in the RTCA assays. To broaden our analysis, we also added the human colon cancer cell line HT29 [24], which is part of the NCI60 human tumour cell line anticancer drug screen panel and therefore well characterized [25]. The LC_50_ for OX in HT29 has been established at 72.44 µM [26]. 

**Figure 5:**
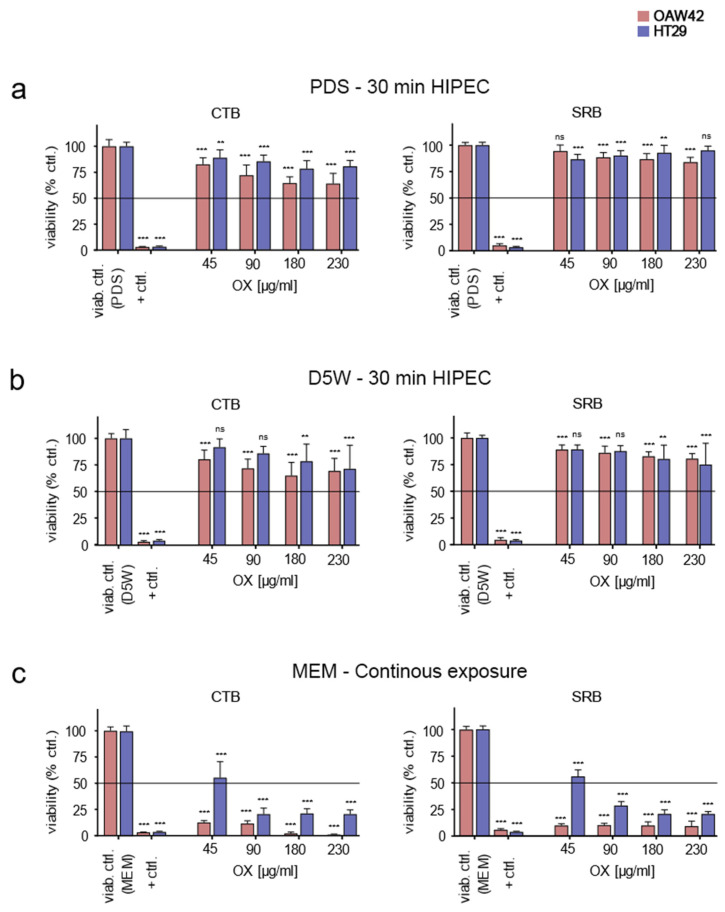
HIPEC simulation for 30 min using prepared OX-containing solutions diluted either in PDS or D5W (CTB and SRB assay). OAW42 cells (red coloration) as well as HT29 cells (blue coloration) were used at a density of 3.15 × 10^5^ cells per well (in a 24-well format) to recreate the conditions encountered in RTCA assays before. HIPEC was simulated for 30 min at 42 °C with prepared solutions containing the specified amounts of OX diluted either in PDS (**a**) or in D5W (**b**). After exposure, cells were washed and subsequently cultured in cell culture medium (MEM) for another 3 days. Further, respective cells were incubated continuously with the specified amounts of OX, spiked into MEM to allow for continuous cell culture and heated likewise (30 min at 42 °C) followed by 72 h cell culture (**c**). Thereafter, the CTB cell viability assay (left graphs) or the SRB cytotoxicity assay (right graphs) were used. Cells were normalized to cells treated identically with D5W, PDS, and MEM only (viab. ctrl.). Positive control (+ ctrl.) was carried out with 1% (*v*/*v*) Triton X-100. Statistical analysis was performed using the Dunnet’s multiple comparison test, confidence interval 95%. ns: *p* ≥ 0.05; **: *p* < 0.01; ***: *p* < 0.001 vs. the respective viability control. The LC_50_ threshold is marked with a black line. Depiction of mean values with standard deviation from 3 independent experiments, with triplicate values assessed in each experiment performed.

After simulated HIPEC for 30 min with 45–230 µg/mL OX diluted in PDS and subsequently culturing the washed cells in medium, both the OAW42 and the HT29 cell lines showed no reduction of mean cell viability below 50% (LC_50_) in none of the assessed conditions within 3 days after treatment (Figure 5a). This finding applied to both assays performed. Repeating the experiments under identical conditions with OX diluted in D5W solution failed also to induce LC_50_ at 72 h following treatment for all tested concentrations (Figure 5b). In contrast, when OX was spiked into cell culture medium (to allow for extended cultivation) and remained with the cells, both cell lines showed clearly enhanced induction of cell death (Figure 5c). Cell viability reproducibly fell below LC_50_ values within 3 days after exposure to all drug concentrations investigated, with the exception of 45 µg/mL OX in medium when tested on HT29 cells. Findings proved reproducible as the results were comparable for both assays.

### 3.5. Drug Solvents Used for HIPEC Reduce Cell Size

**Figure 6:**
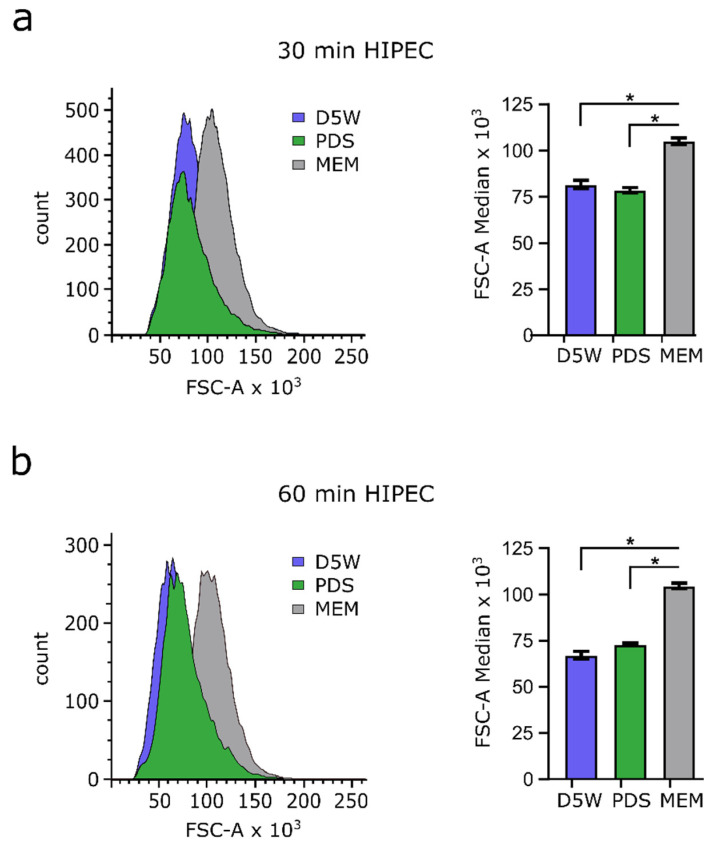
Flow cytometry of OAW42 cells after exposure to different drug diluents for 30 min or 60 min under hyperthermic conditions. OAW42 cells were incubated with D5W (blue coloration) or with PDS (green coloration) at 42 °C and slight shaking (30 rpm) for 30 min (**a**) or 60 min (**b**) and compared to untreated control cells cultured in medium (MEM; grey coloration). Histograms from flow cytometry showing cell counts versus forward scatter area (FSC-A) (left panels) and a comparison of FSC-A between the respective solvents (right panels). Significant differences are marked by an asterisk (*: *p* < 0.05; Bonferroni corrected Student’s *t*-test). Each data point represents the mean value of 3 replicates in independent experiments.

OAW42 cells were exposed to D5W and PDS at 42 °C for either 30 min or 60 min and cell size was measured by flow cytometry, since respective solvents constitute the most frequently used drug diluents for HIPEC with OX. Significant cell size reduction of about 25% (in FSC-A) was observed after 30 min and 60 min of incubation at hyperthermic conditions with both solvents tested (D5W and PDS), when compared to cells maintained in cell culture medium (Figure 6). The observed effects are comparable between both solvents and showed only a slightly more increased shrinkage after 1 h as compared to 30 min exposure.

## 4. Discussion

Over the past decades, survival in metastatic CRC has widely improved. This is also true for metastases limited to the peritoneum, which were considered unresponsive to chemotherapy and therefore a generally palliative disease stage in the past [27]. Here CRS with HIPEC has relevantly enhanced patient prognosis. However, in CRC this complex compound treatment is currently highly controversial, since the randomized controlled PRODIGE 7 trial could not establish survival benefits for patients through adding OX-based HIPEC following CRS [13], whilst increasing morbidity and duration of hospital stay. Further, HIPEC using OX was unsuccessful in an another RCT in the adjuvant setting, where no benefit could be shown for HIPEC to decrease the incidence of metachronous peritoneal metastasis following primary CRC resection [28]. Certainly, it should not go unmentioned that CRS alone was able to increase median overall survival of patients to 41 months according to the PRODIGE 7 trial, which is unprecedented for this disease [13]. These unexpectedly high survival rates in both study cohorts have raised questions pertaining to the study design and the included patient population as well as to tumor biology. These issues include a high rate of crossovers into the HIPEC group and the inclusion of highly selected patients with heavy pretreatment (including systemic OX administration), therefore potentially weakening the validity of these trial results and limiting conclusions that can be drawn regarding HIPEC ineffectiveness [29,30]. In any case, HIPEC with OX currently faces pressure for justification.

Besides the prevailing controversies concerning the clinical use of HIPEC and the interpretation of the PRODIGE 7 trial results, including patient selection and speculations on acquired resistance of peritoneal metastases against OX [14,15,16,31], it should be acknowledged that preclinical research has never been a priority in the implementation of this treatment modality. The consequence of this is an abundance of different HIPEC protocols in clinical use [2,7] and lacking evidence regarding the precise mode of action of HIPEC. In accordance, taking a step back and systematically investigating the effects of the varying treatment parameters of HIPEC in a preclinical setting before implementation in clinical trials is a sensible strategy and should serve as an example [32]. One central but hitherto poorly answered question is the tissue penetration depth of drugs used for HIPEC and more importantly their respective biological effects on cells [17]. Low penetration depth has been identified early on as a potential limiting factor of intraperitoneal drug delivery [33], which was assumed to be a few millimeters at most [34]. While only sparse clinical data are available in this context and in spite of research that suggests an increased penetration depth with heat for OX [35], recent findings suggest ineffectiveness of 30 min OX-based HIPEC in organoids [21]. Therefore, to better assess tissue penetration depth during HIPEC and respective cytotoxic effects in a dedicated 2D model, we repurposed a RTCA assay [18], allowing the continuous assessment of a ~100 µm thick cell layer by using the platinum sensitive cell line OAW42 [20]. For our model we simulated HIPEC conditions by incubating cells with either peritoneal perfusate samples obtained during HIPEC treatment in patients or OX-containing solutions prepared at clinically relevant dosages by dilution in peritoneal dialysis or dextrose solutions [7,13]. Hence, we assume this model can elucidate the direct cytotoxic effects of HIPEC on peritoneal micrometastases. Moreover, it should be mentioned that OX cytotoxicity is well-established for the used cell lines, even at a fraction of the concentrations employed in our experiments [20,36].

The observed results underscore that short-term HIPEC with OX exerts only very slight effects in a model system that requires drugs to penetrate a distance of about 1/10 of a millimeter and to affect exposed cells in this way. A respective lack of effectiveness was witnessed in platinum-sensitive cells after 30 min HIPEC simulation with OX diluted in dextrose solutions over a wide concentration range, also encompassing the drug amounts employed in the PRODIGE 7 trial [13]. According to these results, OX only proved effective when diluted in peritoneal dialysis solution at the highest concentration tested and OX diluted in dextrose solution generally failed to decrease impedance values relevantly, thus suggesting a lack of a profound impact on residual metastatic tissue. Using sample materials obtained during HIPEC from 9 patients and characterized to contain a calculated mean of 93.7 μg/mL OX [18], no relevant effects were observed with materials from 6 patients following 30 min of HIPEC simulation within a measurement window of 72 h. In contrast, prolonging exposure to 1 h relevantly improved cytotoxic effects and proved effective with materials from 7 patients. Further assessment of overall means from different patient samples emphasized lacking effectiveness of 30 min OX-based HIPEC in simulations as well as substantial improvements after prolongation to 60 min, although with large variance underscoring relevant individual differences of the sample materials assessed. 

Recently a study in patient-derived CRC organoids likewise showed that prolonged exposure to OX at lower concentrations was more cytotoxic than short-term exposure at higher concentrations [37]. This notion generally conforms with our results, since the effects observed after simulated HIPEC with materials from 9 patients could be improved by prolonging simulated HIPEC treatment to 60 min. Regarding the RTCA assay with OX-containing solutions prepared at clinically relevant dosages, unfortunately we cannot conclude comprehensively on HIPEC prolongation to 1 h, since with dextrose solution our model proved unsuitable due to cell detachment. Nevertheless, the results from short-term exposure were supported by the assessment of 2 different cell lines in 2 well-established end-point assays using a comparable setting: following 30 min of OX-based HIPEC simulation, less than 25% reduction of cell viability was observed compared to values below 50% cell viability when continuously exposed to OX even at low concentrations. Likewise, long term exposure to OX even at relatively low concentrations unanimously eliminated the exposed cell layer in our RTCA model.

Evidently, as HIPEC involves a variety of factors, these may become relevant in the context of different drugs used and influence molecular mechanisms relevant for anti-cancer effects. Importantly, both the exposure period and heat have been implicated in HIPEC effects. For instance, in vitro studies with OX have shown increased drug uptake and DNA damage with heat, resulting in increased apoptosis but still required 60 min exposure for effectiveness in respective CRC models, whereas, e.g., mitomycin C lacked such synergistic effects [38]. Further, it is known that heat inhibits DNA repair, e.g., through inhibiting poly(ADP-ribose)-polymerase (PARP1), and therefore it can sensitize cells for chemotherapy [39,40]. Another important determinant in HIPEC is the solvent used to dilute the drugs. Here we observed significant cell size reduction of about 25% after 30 min, persisting also at 60 min under hyperthermic conditions with both tested solvents (PDS and D5W as compared to medium). We speculate that the observed changes are most likely caused by a fluid shift to the extracellular space, which also occurs during HIPEC treatment and may impair drug penetration into tissues.

As a limitation of this modelling study it should be acknowledged that this system is artificial, since merely effects on platinum-sensitive cancer cells are assessed and the peritoneal microenvironment cannot be imitated. In reality, increased interstitial fluid pressure in the tumor [41], tissue cohesion, edema formation, and other factors may likely complicate the picture. Using sample materials obtained during HIPEC from patients also has immanent limitations, since, e.g., we found previously that the administered OX does react with contents of peritoneal dialysis solutions by forming new compounds [18], which may affect cellular uptake and molecular drug mechanisms.

Apart from this, the introduced model has several advantages compared to alternative approaches. These include the possibility to investigate HIPEC effects continuously in the 2D model under specific consideration of the penetration depth, which is in contrast to 3D models that show considerably higher degrees of freedom, e.g., with regard to organoid size and other properties [21,37]. Due to the biosensors´ functional assessment a spatial resolution is ensured, informing only about the cell viability of those cells located at the well bottom. Thereby effects on cells can be directly investigated, avoiding the extrapolation of biological effects from drug penetration depth, which itself comes with immanent limitations and idiosyncrasies [42]. Our assay is robust and shows consistency and good reproducibility, allowing for systematic comparisons to be performed. Further continuous measurements provide a good temporal resolution and are therefore more sensitive to changes occurring with delay and superior to end-point assays. Hitherto, there are few good preclinical models for laboratory use and our model is unique in several aspects, adding a new tool to HIPEC research.

Overall, preclinical models for HIPEC are scarce and mainly restricted to animal models so far [43,44], whereas adequate model systems have only been established very recently [21,32,37]. If the findings observed for OX hold true for other tumor entities and drugs used for HIPEC and under normothermic conditions remains unclear, which is why our model system may be a worthwhile addition for future research.

## 5. Conclusions

Overall, our investigations showed severely limited effects of short-term OX-based HIPEC, in line with previous research. We now show that the desired effects could be improved by prolonged exposure. Strikingly, our simulations of HIPEC treatment in platinum-sensitive cell lines using excessive OX dosages proved insufficient to eradicate even minimal tumor cell accumulations. 

Thus, the presented work may support the notion that short-term OX-based HIPEC is ineffective to eliminate peritoneal micrometastasis and may hint to possible causes for this failure. Our findings suggest that drug distribution into deeper tissue layers may be a crucial factor as well as exposure time but also the solvents used for OX dilution in HIPEC may substantially alter drug induced effects.

Based on the restricted penetration depth of most HIPEC drugs [45], systematic preclinical research is warranted. Taken together, HIPEC—in contrast to CRS—remains an experimental approach for treatment of peritoneal metastasis originating from CRC and new evidence is urgently needed.

## Data Availability

The data presented in this study is contained within the article or Supplementary Materials further information is available on request from the corresponding author.

## Supplementary Materials

The following are available online at https://www.mdpi.com/article/10.3390/cancers14051158/s1, Figure S1: Assessment of the thickness of an OAW42 cell layer seeded at different densities; Figure S2: RTCA: Pat. 2: Exposure of OAW42 cells to OCS for 30 min at 42 °C; Figure S3: RTCA: Pat. 2: Exposure of OAW42 cells to OCS for 60 min at 42 °C; Figure S4: RTCA: Pat. 4: Exposure of OAW42 cells to OCS for 30 min at 42 °C; Figure S5: RTCA: Pat. 4: Exposure of OAW42 cells to OCS for 60 min at 42 °C; Figure S6: RTCA: Pat. 5: Exposure of OAW42 cells to OCS for 30 min at 42 °C; Figure S7: RTCA: Pat. 5: Exposure of OAW42 cells to OCS for 60 min at 42 °C; Figure S8: RTCA: Pat. 6: Exposure of OAW42 cells to OCS for 30 min at 42 °C; Figure S9: RTCA: Pat. 6: Exposure of OAW42 cells to OCS for 60 min at 42 °C; Figure S10: RTCA: Pat. 7: Exposure of OAW42 cells to OCS for 30 min at 42 °C; Figure S11: RTCA: Pat. 7: Exposure of OAW42 cells to OCS for 60 min at 42 °C; Figure S12: RTCA: Pat. 8: Exposure of OAW42 cells to OCS for 30 min at 42 °C; Figure S13: RTCA: Pat. 8: Exposure of OAW42 cells to OCS for 60 min at 42 °C; Figure S14: RTCA: Pat. 9: Exposure of OAW42 cells to OCS for 30 min at 42 °C; Figure S15: RTCA: Pat. 9: Exposure of OAW42 cells to OCS for 60 min at 42 °C; Figure S16: RTCA: Oxaliplatin (OX)-spiked into PDS 60 min at 42 °C; Figure S17: RTCA: Continuous exposure of OAW42 cells to OCS in PDS; Figure S18: RTCA: Continuous exposure of OAW42 cells to OCS in D5W.
