# Supplementary material for: Prolonged Exposure to Oxaliplatin during HIPEC Improves Effectiveness in a Preclinical Micrometastasis Model
Source: Cancers (Basel). 2022 Feb 24;14(5):1158. doi: 10.3390/cancers14051158 (PMC8909393; doi:10.3390/cancers14051158)
Supplement: Supplementary file 1 [file cancers-14-01158-s001.zip › cancers-1560055-supplementary.pdf]

Supplement to:

Prolonged exposure to oxaliplatin during HIPEC improves effectiveness in a preclinical micrometastasis model

Nick Seyfried [1,2,3], Can Yurttas [1,*], Markus Burkard [4], Benedikt Oswald [2], Alexander Tolios [5,6,7], Franziska Herster [2,8], Joseph Kauer [2,9,10,11], Tarkan Jäger [12], Ingmar Königsrainer [1,13], Karolin Thiel [1], Markus Quante [1], Hans-Georg Rammensee [2,9,14], Sascha Venturelli [4,15], Matthias Schwab [9,14,16,17,18], Alfred Königsrainer [1,9,14], Stefan Beckert [1,19], Markus W. Löffler [1,2,9,14,16]

1 University Hospital Tübingen, Department of General, Visceral and Transplant Surgery, Hoppe-Seyler-Str. 3, 72076 Tübingen, Germany
2 University of Tübingen, Interfaculty Institute for Cell Biology, Department of Immunology, Auf der Morgenstelle 15, 72076 Tübingen, Germany
3 Technical University of Munich (TUM), Klinikum rechts der Isar, Department of Surgery, Ismaninger Str. 22, 81675 Munich, Germany
4 University of Hohenheim, Institute of Nutritional Sciences, Department of Nutritional Biochemistry, Garbenstr. 30, 70599 Stuttgart, Germany
5 Medical University of Vienna, Department of Blood Group Serology and Transfusion Medicine, Währinger Gürtel 18-20, 1090 Vienna, Austria
6 Medical University of Vienna, Institute of Vascular Biology and Thrombosis Research, Center for Physiology and Pharmacology, Schwarzspanierstraße 17A, 1090 Vienna, Austria
7 Medical University of Vienna, Institute of Artificial Intelligence, Center for Medical Statistics, Informatics, and Intelligent Systems, Spitalgasse 23, 1090 Vienna, Austria
8 Robert Bosch Hospital, Robert Bosch Center for Tumor Diseases (RBCT), Auerbachstr. 110, 70376 Stuttgart, Germany
9 German Cancer Consortium (DKTK) and German Cancer Research Center (DKFZ) partner site Tübingen, 72076 Tübingen, Germany
10 University Hospital Tübingen, Department of Internal Medicine, German Cancer Consortium (DKTK), Clinical Collaboration Unit Translational Immunology, Otfried-Müller-Str. 10, 72076 Tübingen, Germany
11 University Hospital Heidelberg, Department of Hematology, Oncology, and Rheumatology, Im Neuenheimer Feld 410, 69120 Heidelberg, Germany
12 Paracelsus Medical University, Department of Surgery, Müllner Hauptstraße 48, 5020 Salzburg, Austria
13 Landeskrankenhaus Feldkirch, Department of General, Visceral and Thoracic Surgery, Carinagasse 47, 6800 Feldkirch, Austria
14 Cluster of Excellence iFIT (EXC2180) 'Image-Guided and Functionally Instructed Tumor Therapies', University of Tübingen, 72076 Tübingen, Germany
15 University of Tübingen, Institute of Physiology, Department of Vegetative and Clinical Physiology, Wilhelmstr. 56, 72074 Tübingen, Germany
16 University Hospital Tübingen, Department of Clinical Pharmacology, Auf der Morgenstelle 8, 72076 Tübingen, Germany
17 Dr. Margarete Fischer-Bosch-Institute of Clinical Pharmacology, Auerbachstr. 112, 70376 Stuttgart, Germany
18 Departments of Pharmacy and Biochemistry, University of Tübingen, Auf der Morgenstelle 15, 72076 Tübingen, Germany
19 Department of General and Visceral Surgery, Schwarzwald-Baar Hospital, Klinikstr. 11, 78052 Villingen-Schwenningen, Germany

* Correspondence should be addressed to:

Can Yurttas, University Hospital Tübingen, Department of General, Visceral and Transplant Surgery, Hoppe-Seyler-Str. 3, 72076 Tübingen, Germany. E-mail: can.yurttas@me.uni-tuebingen.de

Table of contents

Suppl. Fig. 1 *Assessment of the thickness of an OAW-42 cell layer seeded at different densities* **S3**

Figure Legends to Suppl. Fig. 2-15 **S4**

Suppl. Fig. 2 *RTCA: Pat. 2: Exposure of OAW42 cells to OCS for 30 minutes at 42 °C* **S5**

Suppl. Fig. 3 *RTCA: Pat. 2: Exposure of OAW42 cells to OCS for 60 minutes at 42 °C* **S5**

Suppl. Fig. 4 *RTCA: Pat. 4: Exposure of OAW42 cells to OCS for 30 minutes at 42 °C* **S6**

Suppl. Fig. 5 *RTCA: Pat. 4: Exposure of OAW42 cells to OCS for 60 minutes at 42 °C* **S6**

Suppl. Fig. 6 *RTCA: Pat. 5: Exposure of OAW42 cells to OCS for 30 minutes at 42 °C* **S7**

Suppl. Fig. 7 *RTCA: Pat. 5: Exposure of OAW42 cells to OCS for 60 minutes at 42 °C* **S7**

Suppl. Fig. 8 *RTCA: Pat. 6: Exposure of OAW42 cells to OCS for 30 minutes at 42 °C* **S8**

Suppl. Fig. 9 *RTCA: Pat. 6: Exposure of OAW42 cells to OCS for 60 minutes at 42 °C* **S8**

Suppl. Fig. 10 *RTCA: Pat. 7: Exposure of OAW42 cells to OCS for 30 minutes at 42 °C* **S9**

Suppl. Fig. 11 *RTCA: Pat. 7: Exposure of OAW42 cells to OCS for 60 minutes at 42 °C* **S9**

Suppl. Fig. 12 *RTCA: Pat. 8: Exposure of OAW42 cells to OCS for 30 minutes at 42 °C* **S10**

Suppl. Fig. 13 *RTCA: Pat. 8: Exposure of OAW42 cells to OCS for 60 minutes at 42 °C* **S10**

Suppl. Fig. 14 *RTCA: Pat. 9: Exposure of OAW42 cells to OCS for 30 minutes at 42 °C* **S11**

Suppl. Fig. 15 *RTCA: Pat. 9: Exposure of OAW-42 cells to OCS for 60 minutes at 42 °C* **S11**

Suppl. Fig. 16 *RTCA: Oxaliplatin (OX)-spiked into PDS 60 minutes at 42 °C* **S12**

Supp. Fig. 17 *RTCA: Continuous exposure of OAW42 cells to OCS in PDS* **S13**

Supp. Fig. 18 *RTCA:* Continuous exposure of OAW42 cells to OCS in D5W **S14**

Supp. Fig. 1 Assessment of the thickness of an OAW42 cell layer seeded at different densities

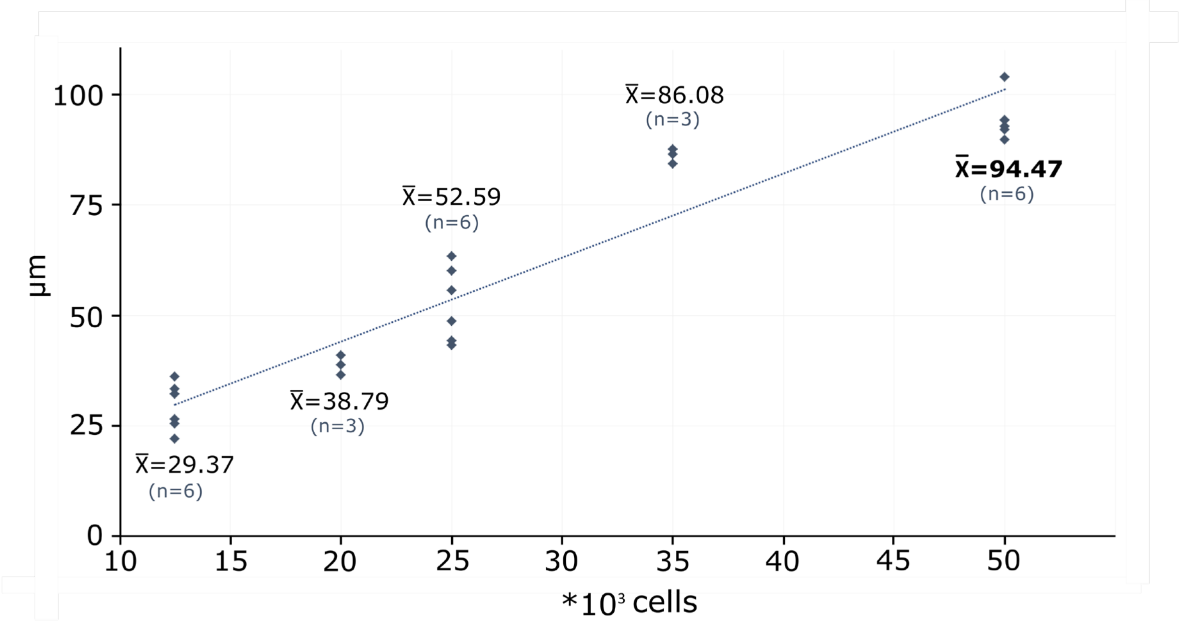

Serial dilutions of OAW42 cells seeded at different densities of 12.5/ 20/ 25/ 35 and 50 x 10^3 cells/ well (in a 96-well plate) were performed. Cell layer thickness was measured in (n) replicates after 24 hours cell culture, using z-stacks on a Nikon Ti Eclipse microscope (performed by an unbiased observer) using 10x magnification with the NIS-Elements (Nikon, Tokyo, Japan) or ImageJ software. Experiments were performed twice to obtain measurements at two independent occasions. Arithmetic means (x̄) of (n) measurements are given in µm.

**Suppl. Fig. 2-15**	**RTCA: Pat. 2 and 4-9: Exposure of OAW42 cells to OCS for 30/ 60 minutes at 42 °C**

Normalized cell index (nCI) in 6-hour intervals from real-time cell analysis (RTCA) impedance measurements of platinum-sensitive OAW42 cells, incubated with oxaliplatin-containing solutions (OCS) (0 h) at 42 °C, previously obtained from patients during HIPEC for the indicated periods and drug solvent circulated through the abdomen sampled before drug application (pre). OCS were obtained at time points 5, 10, 15, 20, 25 and 30 minutes after drug addition to the HIPEC circuit (for Pat. 2 and 4-7) and after 10, 20 and 30 minutes (for Pat. 8 and 9). Controls represent: (+ ctrl.): Triton; (- ctrl.): Physioneal 40 and medium (MEM). Impedance values (nCI) of samples obtained during HIPEC from Pat. 2 and 4-9 incubated for 30 minutes (**Suppl. Fig. 2/ 4/ 6/ 8/ 10/ 12/ 14**, respectively) as well as for 60 minutes (**Suppl. Fig. 3/ 5/ 7/ 9/ 11/ 13/ 15**, respectively) are shown below (page S4 - S10). Patient coding corresponds with Löffler *et al.* (Ann Surg Oncol. 2017; 24(6):1650-1657.) and the sample materials obtained during HIPEC used here are identical to those used previously. Values were normalized to 1 at the start of treatment (0 h). A decrease of nCI values signifies cell death of OAW42 target cells. Graphs show mean ± SD (of 2-6 technical replicates).

Suppl. Fig. 2 RTCA: Pat. 2: Exposure of OAW-42 cells to OCS for 30 minutes at 42 °C

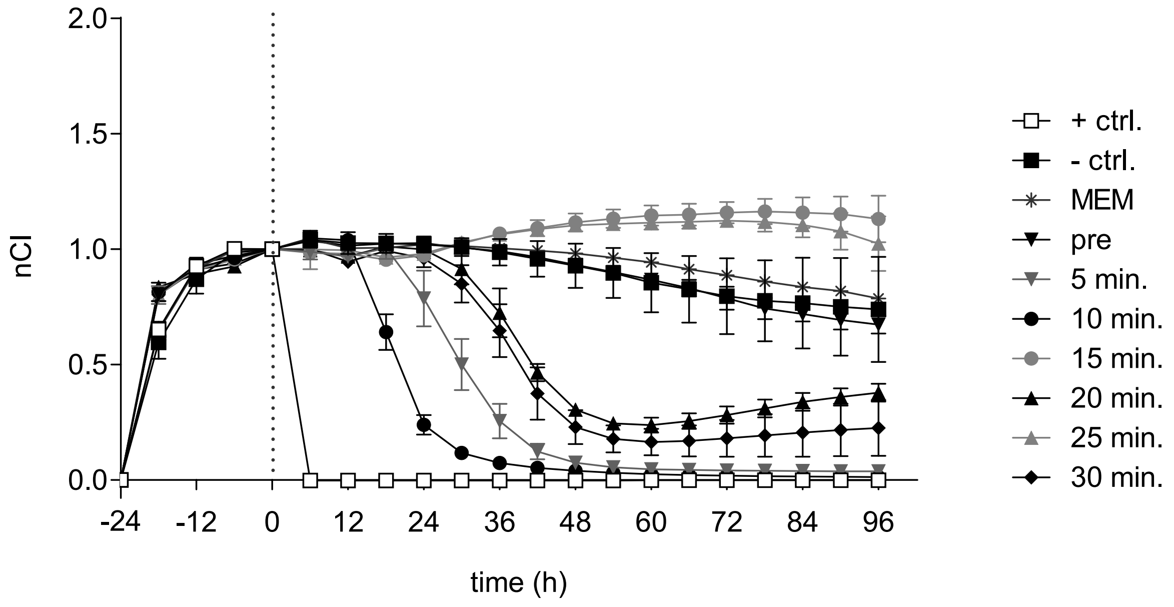

Suppl. Fig. 3 RTCA: Pat. 2: Exposure of OAW-42 cells to OCS for 60 minutes at 42 °C

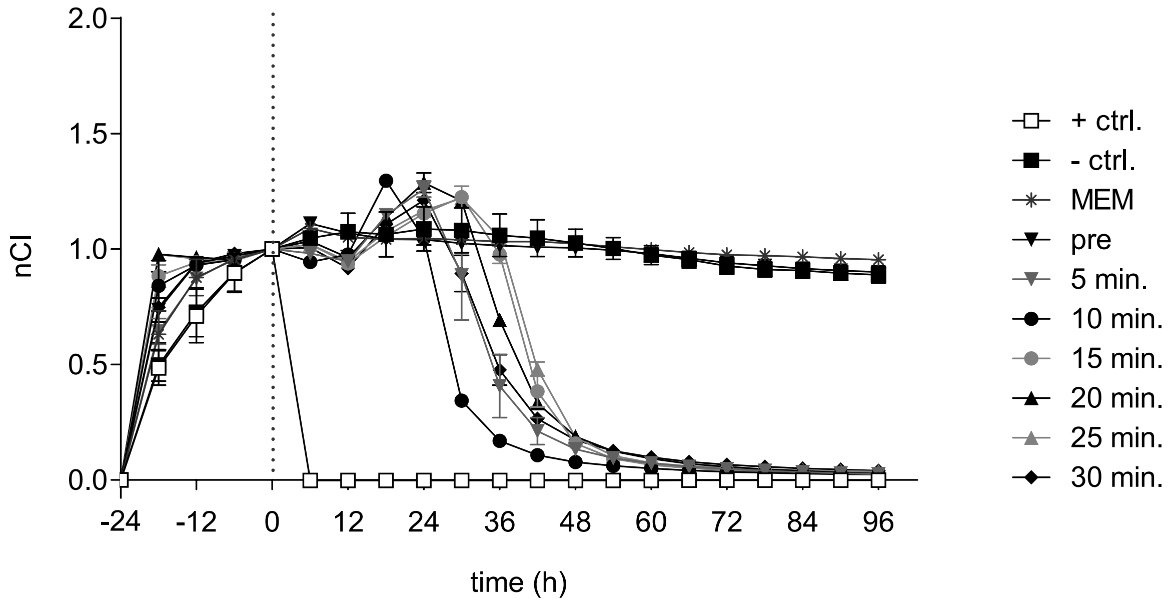

Suppl. Fig. 4 RTCA: Pat. 4: Exposure of OAW-42 cells to OCS for 30 minutes at 42 °C

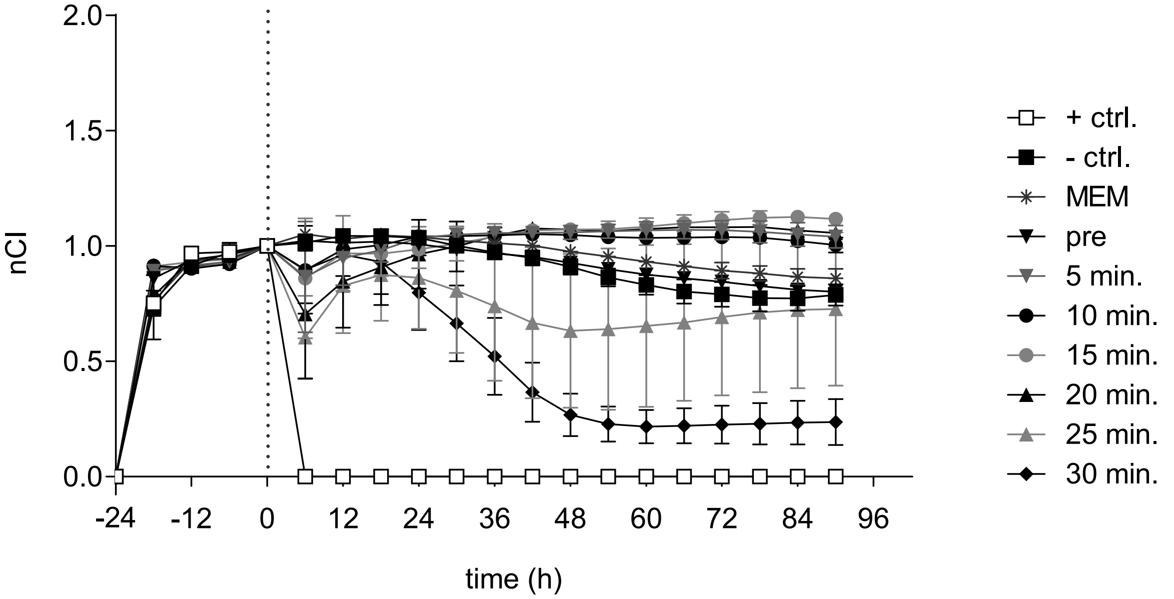

Suppl. Fig. 5 RTCA: Pat. 4: Exposure of OAW42 cells to OCS for 60 minutes at 42 °C

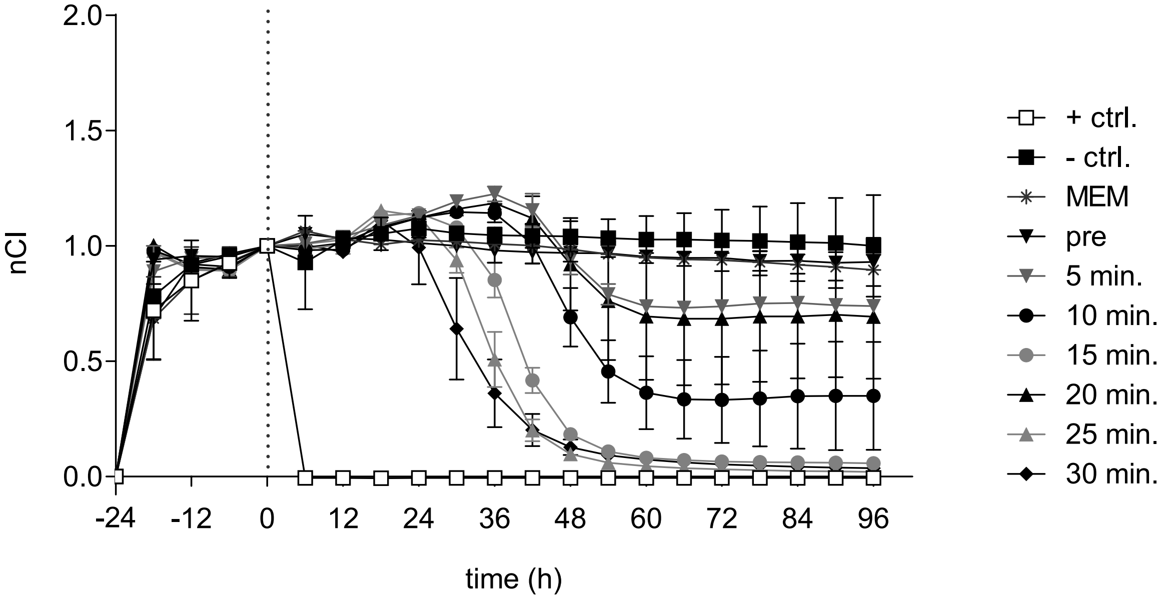

Suppl. Fig. 6 RTCA: Pat. 5: Exposure of OAW42 cells to OCS for 30 minutes at 42 °C

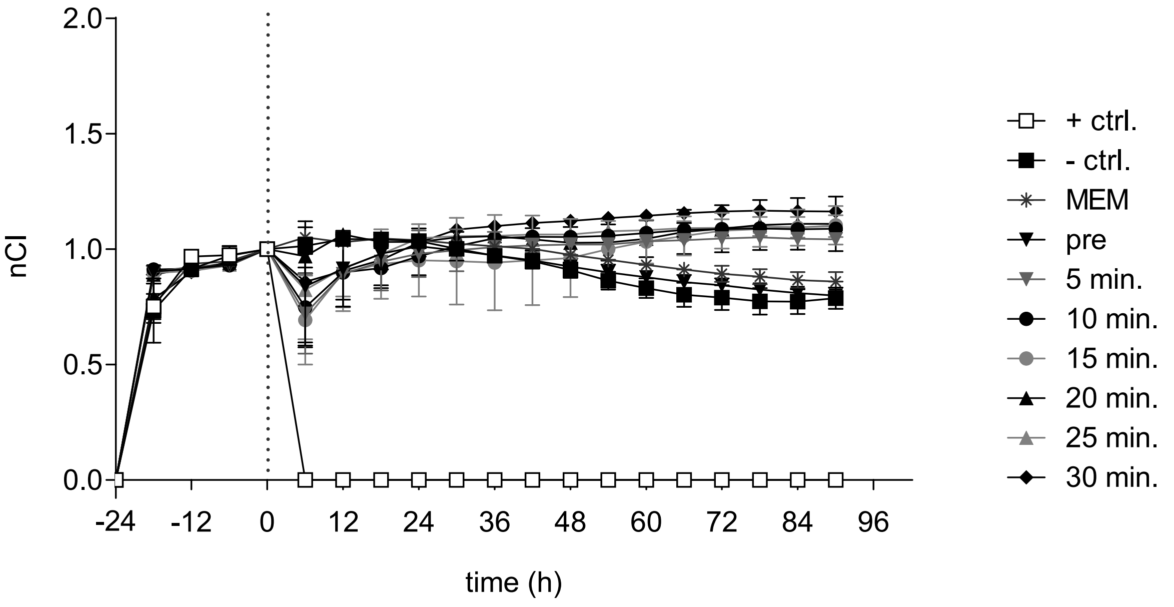

Suppl. Fig. 7 RTCA: Pat. 5: Exposure of OAW42 cells to OCS for 60 minutes at 42 °C

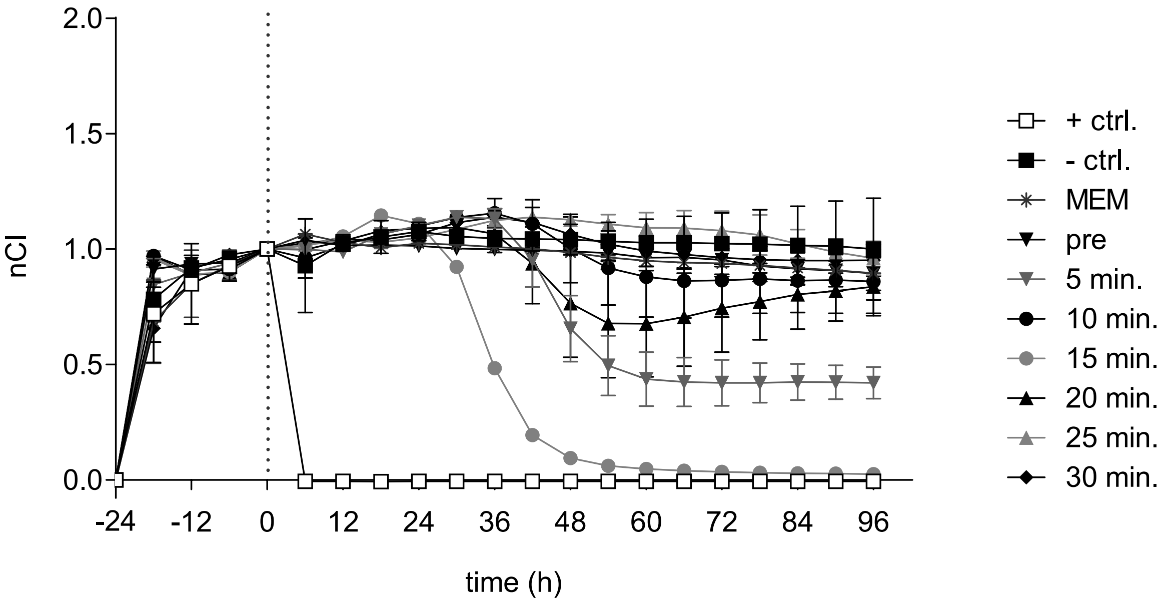

Suppl. Fig. 8 RTCA: Pat. 6: Exposure of OAW42 cells to OCS for 30 minutes at 42 °C

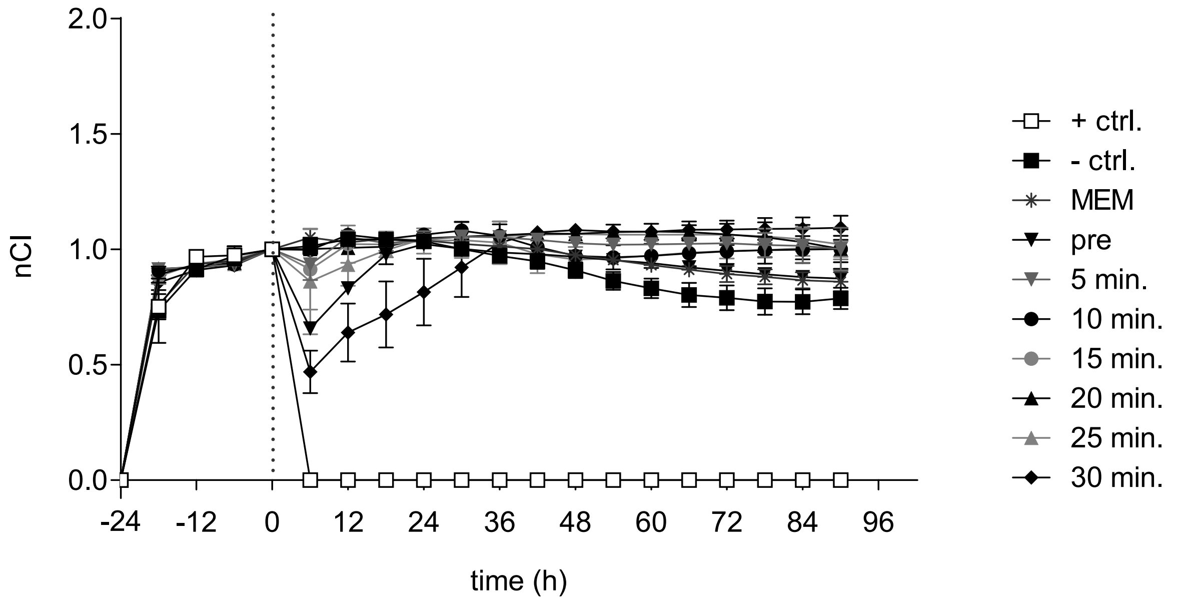

Suppl. Fig. 9 RTCA: Pat. 6: Exposure of OAW42 cells to OCS for 60 minutes at 42 °C

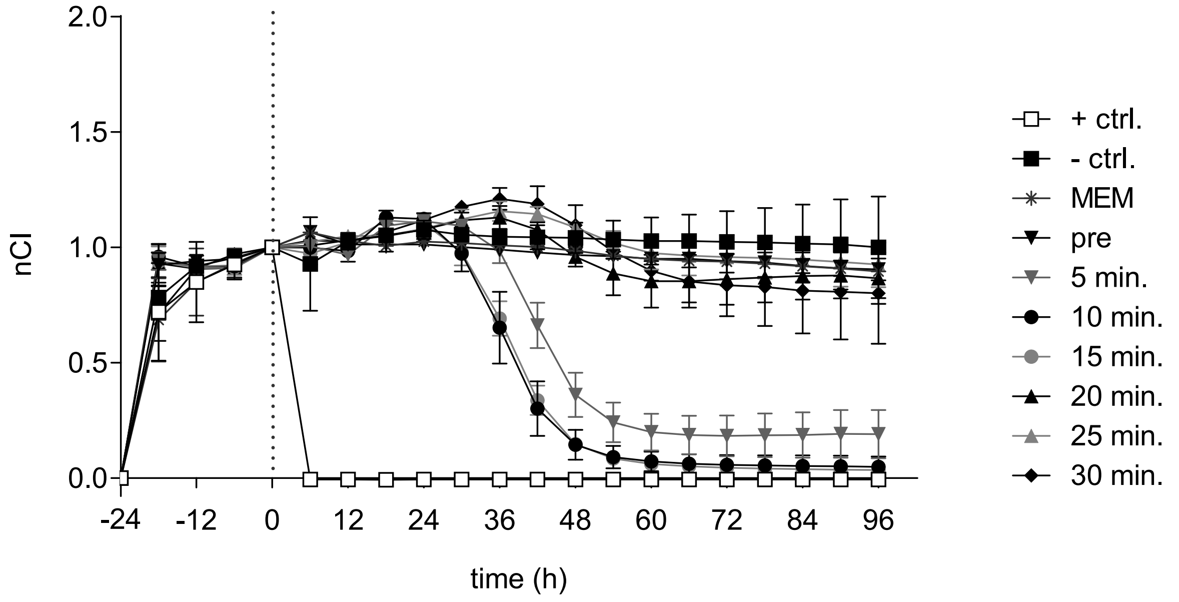

Suppl. Fig. 10 RTCA: Pat. 7: Exposure of OAW42 cells to OCS for 30 minutes at 42 °C

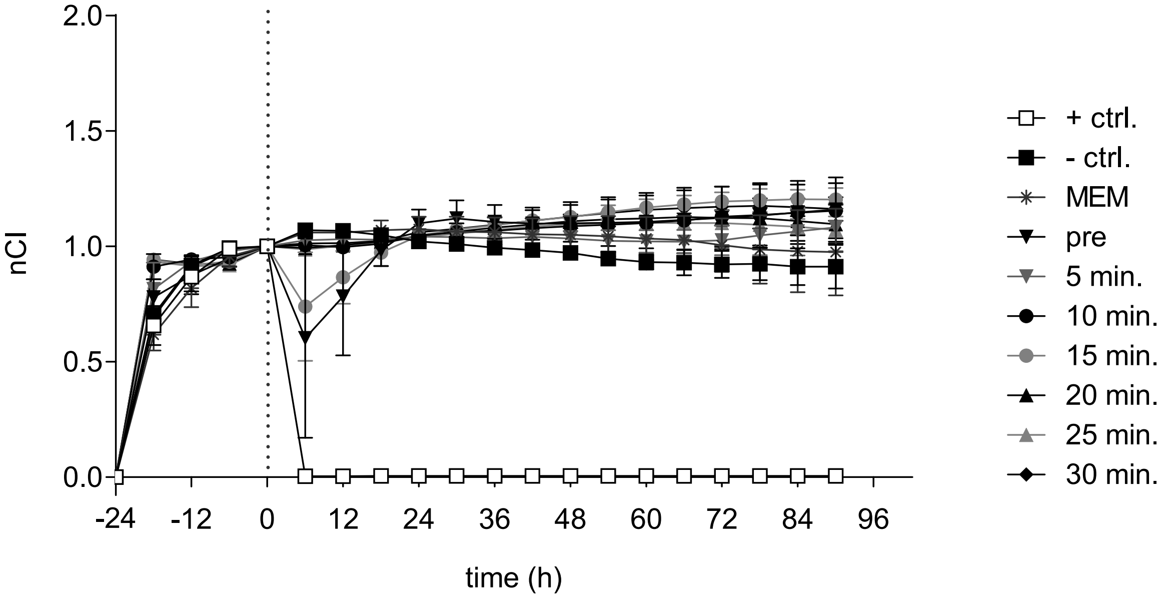

Suppl. Fig. 11 RTCA: Pat. 7: Exposure of OAW42 cells to OCS for 60 minutes at 42 °C

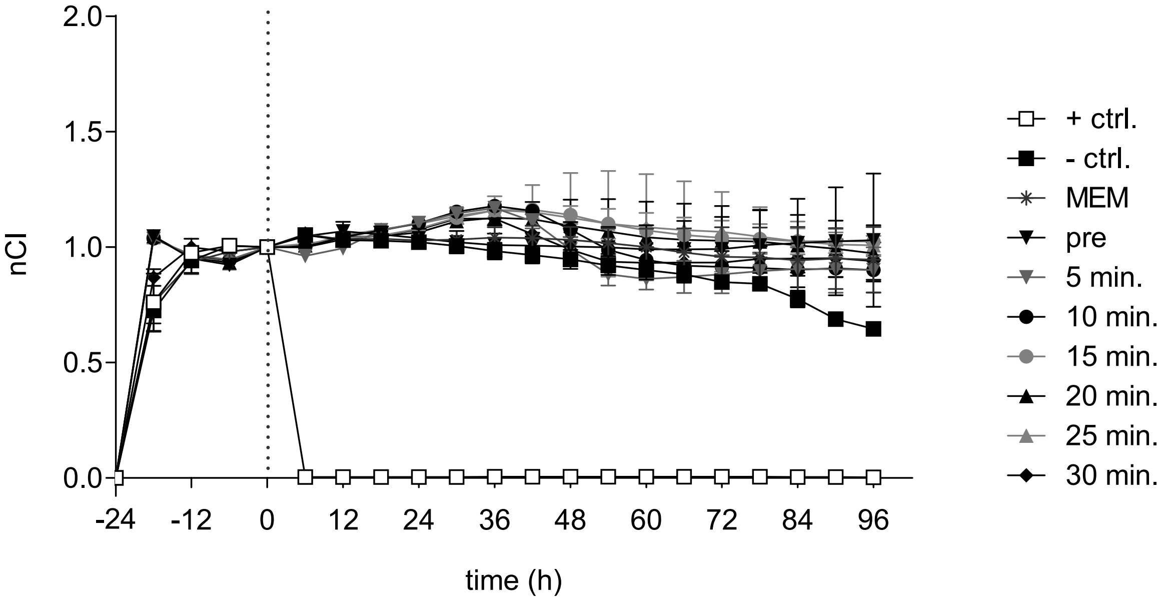

Suppl. Fig. 12 RTCA: Pat. 8: Exposure of OAW42 cells to OCS for 30 minutes at 42 °C

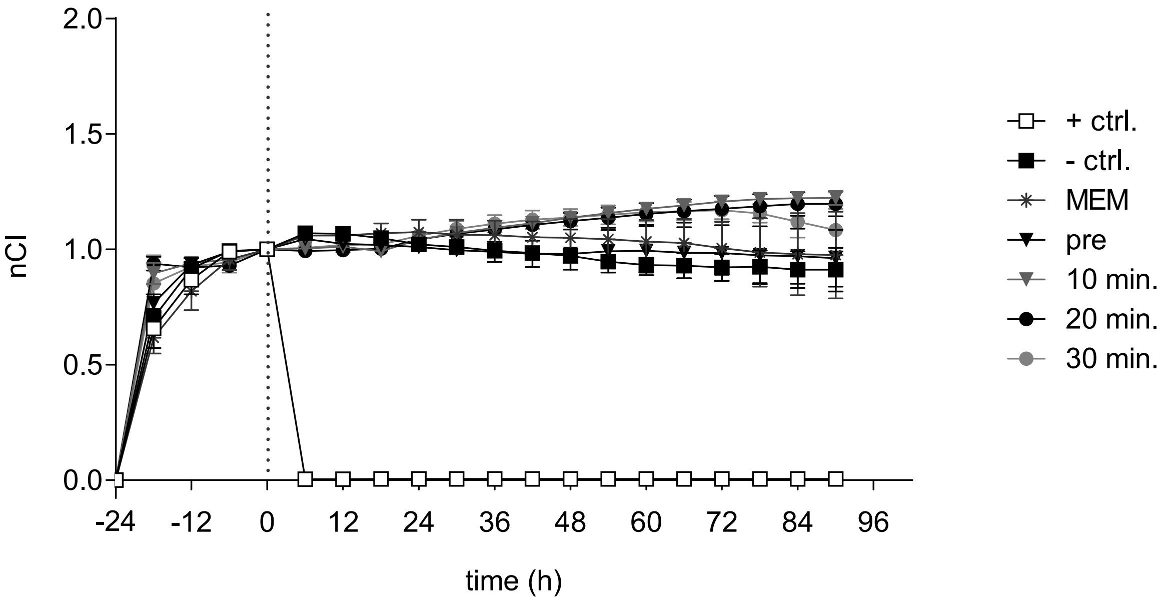

Suppl. Fig. 13 RTCA: Pat. 8: Exposure of OAW42 cells to OCS for 60 minutes at 42 °C

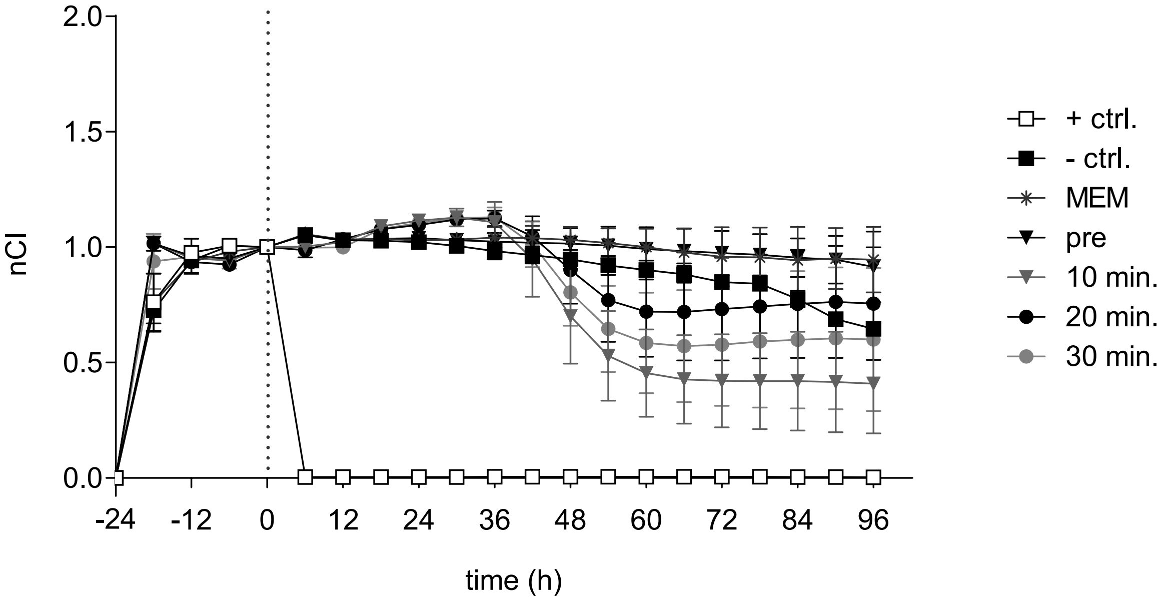

Suppl. Fig. 14 RTCA: Pat. 9: Exposure of OAW42 cells to OCS for 30 minutes at 42 °C

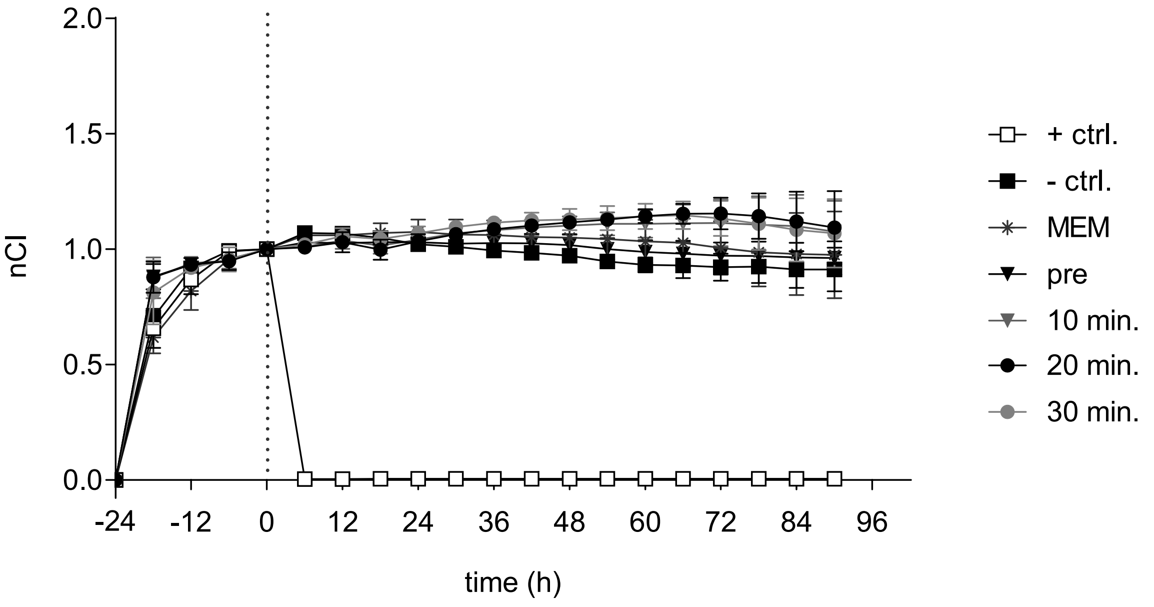

Suppl. Fig. 15 RTCA: Pat. 9: Exposure of OAW42 cells to OCS for 60 minutes at 42 °C

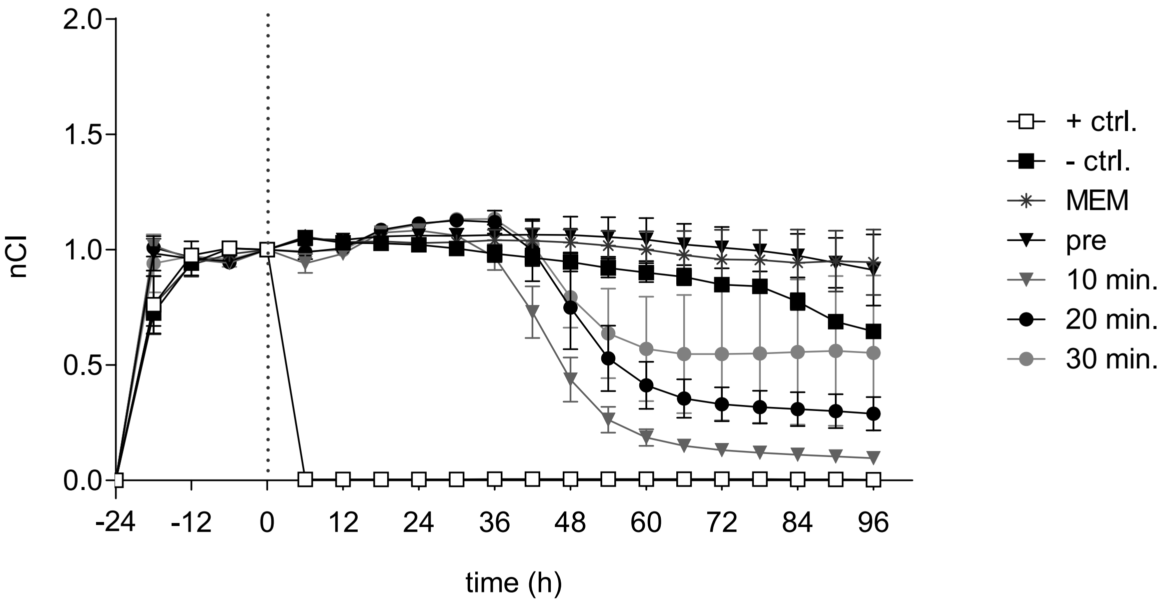

Supp. Fig. 16 RTCA: Oxaliplatin (OX)-spiked into PDS 60 minutes at 42 °C

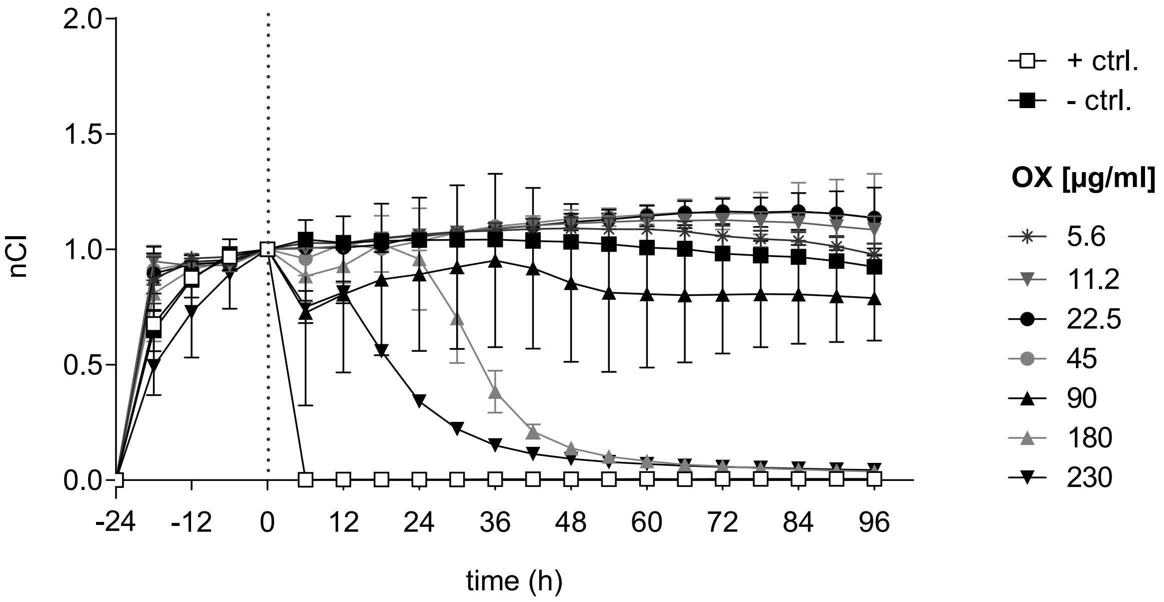

Normalized cell index (nCI) in 6-hour intervals obtained from RTCA impedance measurements of platinum-sensitive OAW42 cells incubated for 60 minutes at 42 °C with the specified amounts of OX spiked into PDS performed at time point 0 hours (h). (+ ctrl.): Triton; (- ctrl.): Physioneal 40; OCS (oxaliplatin-containing solutions); OX (oxaliplatin); PDS (peritoneal dialysis solution; Physioneal 40); RTCA (real-time cell analysis). Graphs show mean ± SD (of 2-3 technical replicates).

Supp. Fig. 17 RTCA: Continuous exposure of OAW42 cells to OCS in PDS

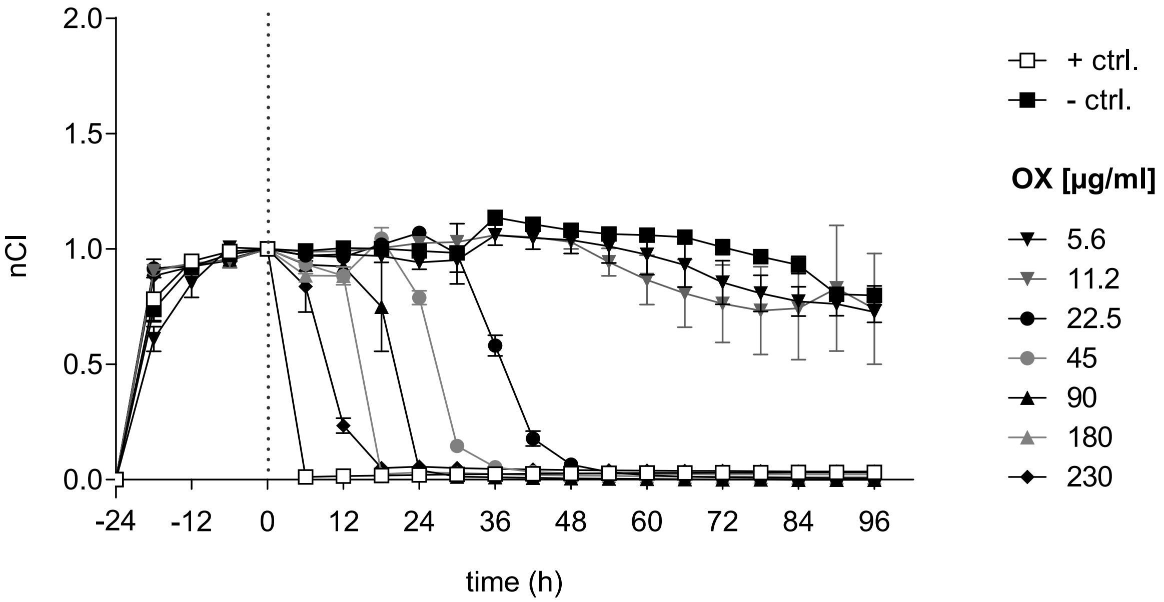

Normalized cell index (nCI) in 6-hour intervals obtained from RTCA impedance measurements of platinum-sensitive OAW42 cells incubated with the specified concentrations of OX initially spiked into PDS and diluted in MEM (effective end concentrations of OX after dilution in 50 % MEM are given) performed and normalized to 1 at time point 0 hours (h). (+ ctrl.): Triton; (- ctrl.): Physioneal 40. MEM (serum-supplemented cell culture medium); OCS (oxaliplatin-containing solutions); OX (oxaliplatin); PDS (peritoneal dialysis solution; Physioneal 40); RTCA (real-time cell analysis). Graphs show mean ± SD (of 2-6 technical replicates).

Supp. Fig. 18 RTCA: Continuous exposure of OAW42 cells to OCS in D5W

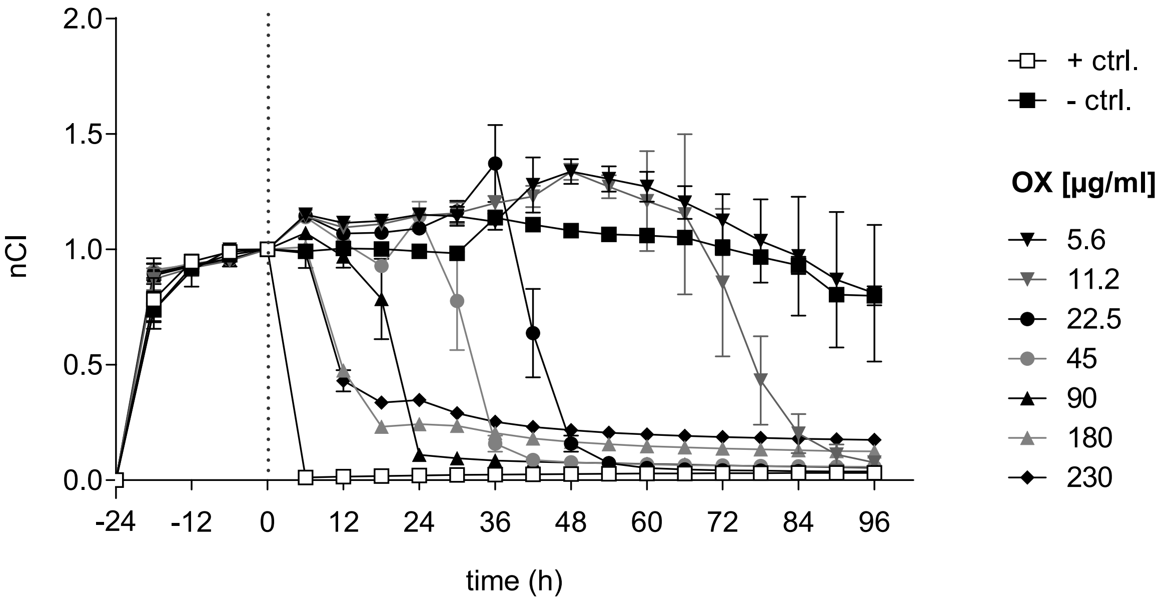

Normalized cell index (nCI) in 6-hour intervals obtained from RTCA impedance measurements of platinum-sensitive OAW42 cells incubated with the specified concentrations of OX spiked into D5W and diluted in MEM (effective end concentrations of OX after dilution in 50 % MEM are given) performed and normalized to 1 at time point 0 hours (h). (+ ctrl.): Triton; (- ctrl.): D5W: dextrose 5 % in water. MEM (serum-supplemented cell culture medium); OCS (oxaliplatin-containing solutions); OX (oxaliplatin); RTCA (real-time cell analysis). Graphs show mean ± SD (of 2-6 technical replicates).
